# Folic acid induces cell type-specific changes in the transcriptome of breast cancer cell lines: a proof-of-concept study

**DOI:** 10.1017/jns.2016.8

**Published:** 2016-04-26

**Authors:** R. Jordan Price, Karen A. Lillycrop, Graham C. Burdge

**Affiliations:** 1 Academic Unit of Human Development and Health, Faculty of Medicine, University of Southampton, Southampton, UK; 2 Centre for Biological Sciences, Faculty of Natural and Environmental Sciences, University of Southampton, Southampton, UK

**Keywords:** Folic acid, Folate transporters, Cell proliferation, Breast cancer, Microarrays, Folate receptors, Pathway analysis, 5mTHF, 5-methyl tetrahydrofolate, BC, breast cancer, FA, folic acid

## Abstract

The effect of folic acid (FA) on breast cancer (BC) risk is uncertain. We hypothesised that this uncertainty may be due, in part, to differential effects of FA between BC cells with different phenotypes. To test this we investigated the effect of treatment with FA concentrations within the range of unmetabolised FA reported in humans on the expression of the transcriptome of non-transformed (MCF10A) and cancerous (MCF7 and Hs578T) BC cells. The total number of transcripts altered was: MCF10A, seventy-five (seventy up-regulated); MCF7, twenty-four (fourteen up-regulated); and Hs578T, 328 (156 up-regulated). Only the cancer-associated gene *TAGLN* was altered by FA in all three cell lines. In MCF10A and Hs578T cells, FA treatment decreased pathways associated with apoptosis, cell death and senescence, but increased those associated with cell proliferation. The folate transporters SLC19A1, SLC46A1 and FOLR1 were differentially expressed between cell lines tested. However, the level of expression was not altered by FA treatment. These findings suggest that physiological concentrations of FA can induce cell type-specific changes in gene regulation in a manner that is consistent with proliferative phenotype. This has implications for understanding the role of FA in BC risk. In addition, these findings support the suggestion that differences in gene expression induced by FA may involve differential activities of folate transporters. Together these findings indicate the need for further studies of the effect of FA on BC.

Folic acid (FA) is the synthetic form of folate that is used in nutritional supplements and food fortification. FA is chemically more stable and has a higher bioavailability than food folates^(^^1^^)^. It is uncertain whether folate has a beneficial or adverse effect on the development and progression of breast cancer (BC)^(^^2^^–^^6^^)^. However, the relatively few studies that report the specific effect of FA on BC risk, as opposed to total intake of folate, show a consistent negative effect of higher FA intakes. Women who took FA supplements containing ≥400 µg FA/d showed a 19 % increase in BC risk compared with those who did not take supplements^(^^7^^)^. A case–control study showed a 53 % increase in BC risk in women who took FA supplements compared with those who did not^(^^8^^)^. Furthermore, animal models of chemically induced BC have shown that feeding a diet with a suboptimal FA content suppressed tumorigenesis, possibly by limiting capacity for DNA synthesis and cell division^(^^9^^,^^10^^)^. Conversely, FA supplementation in rats has been shown to enhance the induction, early promotion, and the progression of mammary tumorigenesis in some^(^^9^^,^^11^^)^, but not all^(^^10^^)^, studies. Such findings are of concern because of the high prevalence of multivitamin use in women with BC or BC survivors^(^^12^^,^^13^^)^.

Part of the apparent differential effects of FA and folate on BC may be due to differences in their metabolism. FA and folates that are absorbed by enterocytes are converted to 5-methyl tetrahydrofolate (5mTHF) which is the main chemical form in blood, and which is an important co-factor in one-carbon metabolism and nucleotide biosynthesis^(^^14^^)^. FA intakes of ≥400 µg/d may result in unmetabolised FA in blood in addition to higher 5mTHF concentration^(^^15^^)^ due to the lower capacity of enterocytes to convert FA to 5mTHF compared with folate^(^^16^^)^. It is uncertain whether unmetabolised FA induces adverse effects on health^(^^17^^)^, although it has been associated with impaired natural killer cell activity in women which may have implications for BC risk^(^^18^^)^. Furthermore, since 5mTHF is involved in the supply of methyl groups to the remethylation cycle in which DNA is a terminal acceptor, FA may alter BC risk through altered epigenetic regulation of tumour suppressor genes as BC can involve hypermethylation of P16INK4a, BRCA1, BRCA2, oestrogen receptor (ER)-α, retinoic acid receptor-β2, APC, and RASSF1A promoters leading to lower transcription and to impaired DNA repair^(^^19^^–^^21^^)^.

5mTHF and unmetabolised FA can be assimilated by cells via either folate receptors, reduced folate carrier protein (RFCP, *SCL19A1*) or the proton-coupled folate transporter (PCFT, *SCL46A1*)^(^^22^^)^. Each of these transporters exhibits a cell type-specific distribution and more than one transporter may be present in each cell type^(^^23^^–^^25^^)^. Two folate transporters have been associated with cancer outcomes. Folate receptor-α (FRα, *FOLR1*) expression has been shown to be increased in oestrogen receptor (ER) and progesterone receptor (PR) triple negative BC cells and is associated with poor prognosis^(^^26^^)^, while RFCP has been shown to be differentially expressed in B-cell lymphoma^(^^23^^)^. Furthermore, the affinity of these folate transporters for FA is 5- to 10-fold greater than for 5mTHF^(^^27^^)^. Thus differences in capacity for FA transport between BC cell types may be one important factor that determines the effect of FA on cancer-related processes and so may contribute to the variation in the outcomes of studies of the association between folate and FA intakes or status and BC risk.

To understand better the effect of FA on BC, we investigated the effect of treating non-transformed breast and BC cell lines with FA concentrations within the range of unmetabolised FA that has been reported previously in blood of humans taking FA supplements^(^^28^^)^ and carried out transcriptome-wide analysis using microarray. We also investigated whether FA receptor expression differed between these cell lines.

## Methods

### Folic acid treatment of cell lines

The three human cell lines used in this study were chosen for their differences in phenotype. The non-transformed MCF10A cells acted as a non-tumorigenic control, while MCF7 and Hs578T cells represented ER-positive and PR-positive, and triple-negative breast adenocarcinoma tumours, respectively. MCF10A human non-transformed breast epithelial cells were obtained from American Type Culture Collection. Hs578T human cells were obtained from the European Collection of Cell Cultures. MCF7 human cells were from our archive, which were derived originally from cells purchased from the European Collection of Cell Cultures. All cell lines were cultured at 37°C in an atmosphere containing 5 % (v/v) CO_2_, in Dulbecco's modified Eagle's medium without FA (Sigma), supplemented with 10 % (v/v) fetal bovine serum (FBS), 2 mm-glutamine, 10 U/ml penicillin and 100 µg/ml streptomycin. The media for the MCF10A cell line was further supplemented with 20 ng/ml epidermal growth factor and 100 µg/ml hydrocortisone. Concentrations of unmetabolised FA up to 273 nmol/l have been reported in serum from individuals consuming ≥400 µg FA/d^(^^29^^)^. Cultures were treated with 0 or 100 nmol/l FA for 72 h (*n* 6 replicates/treatment), the period that allowed maximum cell yield while maintaining the cultures in a subconfluent state. Background folate concentration derived from FBS in the medium was 1·5 nmol/l.

### Microarray analysis of gene expression

At the end of the treatment period, cells were harvested using TRI Reagent (Sigma) and total RNA was extracted^(^^30^^)^. The RNA was further purified using an RNeasy MinElute Cleanup Kit (Qiagen) according to the manufacturer's instructions. RNA concentration and purity were measured using a Nanodrop ND-1000, and RNA integrity was assessed using an Agilent 2100 Bioanalyzer (Agilent Technologies). In all cases the absorbance ratios at 260 and 280 nm were greater than 2 and RNA integrity number scores were above 7. Gene expression profiles were determined using an Illumina HumanHT-12 v4 Expression BeadChip microarray (47 231 probes per sample) carried out by Barts and the London Genome Centre (London, UK), in accordance with the company's quality-control procedures using standard protocols for labelling, hybridisation and washing. The BeadChips were scanned using an Illumina BeadArray Reader and the data were quintile normalised in Illumina BeadStudio. A list of differentially expressed transcripts was generated using cut-offs of *P* value <0·05 and a fold change of at least 1·2 in either direction. Ingenuity Pathway Analysis (IPA, Qiagen) software was used to identify functions and molecules that were predicted to be altered based on the differentially expressed transcripts. All reported analyses from IPA had z-scores with *P* < 0·05. Multiexperiment Viewer (MeV; TM4 Microarray Software Suite) was used to visualise the significantly altered transcripts from each cell line alongside the corresponding transcripts in the other cell lines.

### Quantitative RT-PCR

Measurement of mRNA expression was carried out essentially as described previously^(^^31^^)^. Briefly, complementary DNA was prepared from 1 µg of the same RNA as used for the microarray using Moloney-murine leukaemia virus reverse transcriptase (Promega). Quantitative RT-PCR was performed in a total reaction volume of 10 µl with SYBR Green JumpStart Taq ReadyMix (Sigma) and QuantiTect primer assays (Table 1; Qiagen). mRNA levels were determined by the standard curve method^(^^32^^)^ and normalised to glyceraldehyde 3-phosphate dehydrogenase (*GAPDH*) expression, which was found to be unaffected in all three cell lines. All samples were analysed in duplicate.

**Table 1.** Quantitative RT-PCR primer assays*

Transcript	QuantiTect assay
*ASMTL*	QT00097720
*FOLR1*	QT01000853
*GAPDH*	QT00079247
*MXRA8*	QT00202790
*NMB*	QT00066745
*PHF5A*	QT00080360
*SLC19A1*	QT00014742
*SLC25A4*	QT00048279
*SLC46A1*	QT00067515
*UBE2J1*	QT00029855

* Primers were QuantiTect assays purchased from Qiagen. Primer sequences were not available from the company.

### Statistical analysis

Statistical analyses were carried out using SPSS (v21; IBM Corporation). An unpaired Student's *t* test was used to analyse the differences between the control and treated microarray results. Folate transporter data were analysed by two-way ANOVA using Bonferroni's *post hoc* correction with treatment and cell line as fixed factors. Differences were considered to be statistically significant at *P* < 0·05.

## Results

### Validation of microarray by quantitative RT-PCR

To validate the microarray data, quantitative RT-PCR was performed on four randomly selected transcripts, with varying magnitudes and directions of change, for each of the three cell lines. In these three cell lines, 11/12 transcripts measured by quantitative RT-PCR had the same direction of change in expression as found with the microarray data (Table 2). *PHF5A* in the Hs578T cells showed no change (Table 2).

**Table 2.** Validation of microarray analysis by quantitative RT-PCR (qRT-PCR)

Gene symbol	Microarray (fold change)	qRT-PCR (fold change)
MCF10A		
* ASMTL*	−1·11	−1·10
* NMB*	1·08	1·28
* SLC19A1*	−1·01	−1·07
* SLC25A4*	1·09	1·12
MCF7		
* NMB*	1·15	1·27
* PHF5A*	1·12	1·18
* SLC25A4*	−1·08	−1·31
* UBE2J1*	1·13	1·17
Hs578T		
* ASMTL*	−1·08	−1·06
* MXRA8*	−1·50	−1·34
* PHF5A*	1·02	1·00
* SLC25A4*	−1·06	−1·05

### Folic acid induces differential transcriptome changes in different breast cancer cell lines

Cut-offs of *P* value <0·05 and a fold change of at least 1·2 in either direction were used to generate a list of differentially expressed transcripts. These data have been deposited in NCBI's Gene Expression Omnibus^(^^33^^)^ and are accessible through GEO Series accession number GSE68651 (http://www.ncbi.nlm.nih.gov/geo/query/acc.cgi?acc=GSE68651).

The mRNA expression of seventy-five transcripts differed significantly between the control and treated MCF10A cells (seventy up-regulated, five down-regulated). Treatment with FA in the MCF7 cells induced altered expression in a total of twenty-four transcripts compared with the control group (fourteen up-regulated, ten down-regulated). In the Hs578T cells, FA treatment induced altered expression of 328 transcripts (156 up-regulated, 172 down-regulated). Details of the differentially expressed transcripts from MCF10A, MCF7 and Hs578T cell lines are reported in Supplementary Tables S1, S2 and S3, respectively.

**Fig. 1.**
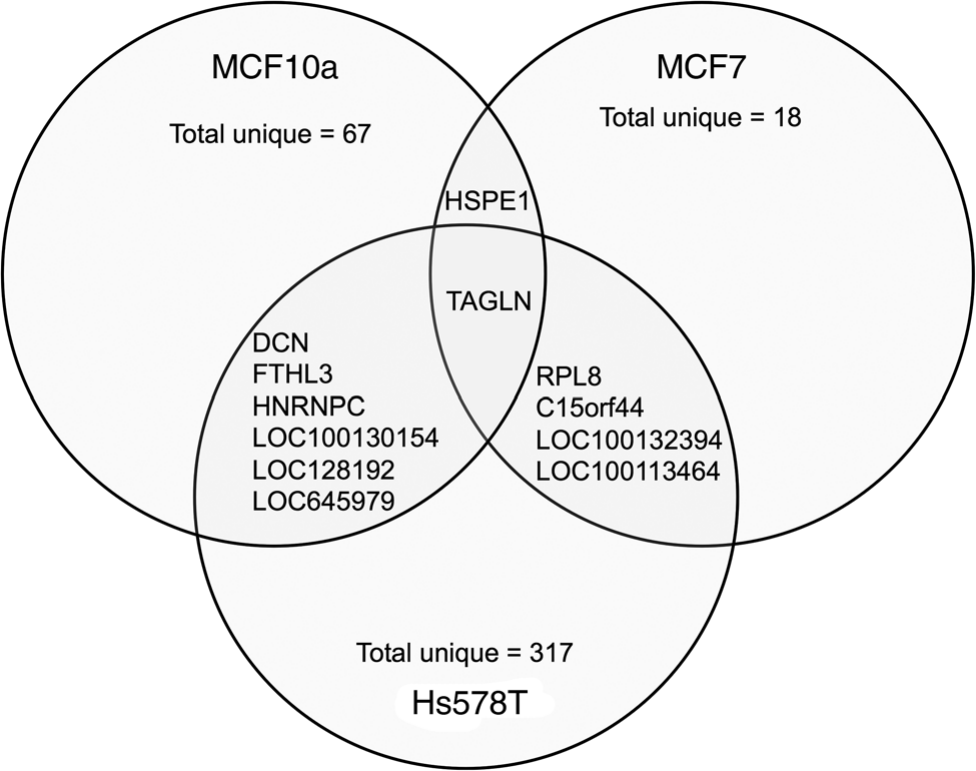
Transcripts altered by folic acid (FA) treatment. Comparative analysis of significantly altered transcripts from each cell line (MCF10a, MCF7 and Hs578T) after FA treatment. The overlaps between the circles indicate the transcripts altered in more than one cell line. The identities of these transcripts and the magnitude of difference are detailed in Table 3.

The transcripts that were altered significantly in each cell line were visualised using a heatmap against the corresponding transcripts from the other cell lines (Supplementary Fig. S1). Comparative analysis showed that the majority of transcripts with altered expression in response to FA were unique to each cell line; MCF10A (89 %), MCF7 (75 %) and Hs578T (97 %) (Fig. 1). FA treatment increased *HSPE1* expression in both MCF10A and MCF7 cells (Table 3). Six transcripts had altered expression in both MCF10A and Hs578T cells (Fig. 1). *DCN*, *FTHL3*, LOC100130154, LOC128192 and LOC645979 expression was increased in MCF10A cells, but decreased expression in Hs578T cells (Table 3). However, expression of *HNRNPC* was up-regulated in both MCF10A and Hs578T cell lines (Table 3). Four transcripts were altered in both MCF7 and Hs578T cells (Fig. 1(B)). Expression of *RPL8* and *C15orf44* was increased in FA-treated MCF7 cells, but decreased in Hs578T cells. Expression of *LOC100132394* and *LOC100134364* was decreased in FA-treated MCF7 cells, but increased in Hs578T cells (Table 3). Only one transcript (*TAGLN*) was altered by FA treatment in all three cell lines (Fig. 1). Expression of *TAGLN* was decreased in MCF10A and Hs578T cells, but increased in MCF7 cells (Table 3).

**Table 3.** Transcripts with significantly altered expression in more than one cell line

Gene	Gene description	MCF10A (fold change)	MCF7 (fold change)	Hs578T (fold change)
*TAGLN*	*Homo sapiens* transgelin (TAGLN), transcript variant 2, mRNA	−1·25	1·21	−1·26
*HSPE1*	*Homo sapiens* heat shock 10 kDa protein 1 (chaperonin 10) (HSPE1), mRNA	1·46	1·54	–
*DCN*	*Homo sapiens* decorin (DCN), transcript variant A1, mRNA	1·29	–	−1·26
*FTHL3*	*Homo sapiens* ferritin, heavy polypeptide-like 3 (FTHL3), non-coding RNA	1·27	–	−1·28
*HNRNPC*	*Homo sapiens* heterogeneous nuclear ribonucleoprotein C (C1/C2) (HNRNPC), transcript variant 3, mRNA	1·20	–	1·25
*LOC100130154*	PREDICTED: *Homo sapiens* similar to thymosin, β10 (LOC100130154)	1·26	–	−1·22
*LOC128192*	PREDICTED: *Homo sapiens* hypothetical LOC128192 (LOC128192)	1·34	–	−1·30
*LOC645979*	PREDICTED: *Homo sapiens* similar to ribosomal protein S26 (LOC645979), mRNA	1·22	–	−1·28
*RPL8*	*Homo sapiens* ribosomal protein L8 (RPL8), transcript variant 2, mRNA	–	1·29	−1·35
*C15orf44*	*Homo sapiens* chromosome 15 open reading frame 44 (C15orf44), transcript variant 2, mRNA	–	1·22	−1·24
*LOC100132394*	PREDICTED: *Homo sapiens* hypothetical protein LOC100132394 (LOC100132394), mRNA	–	−1·48	2·00
*LOC100134364*	PREDICTED: *Homo sapiens* hypothetical protein LOC100134364 (LOC100134364), mRNA	–	−1·43	1·72

### Pathway analysis of transcripts altered by folic acid treatment in MCF10A and Hs578T cells

We were unable to carry out pathway analysis of the effect of FA treatment on MCF7 cells because of the small number of transcripts that showed altered expression.

**Fig. 2.**
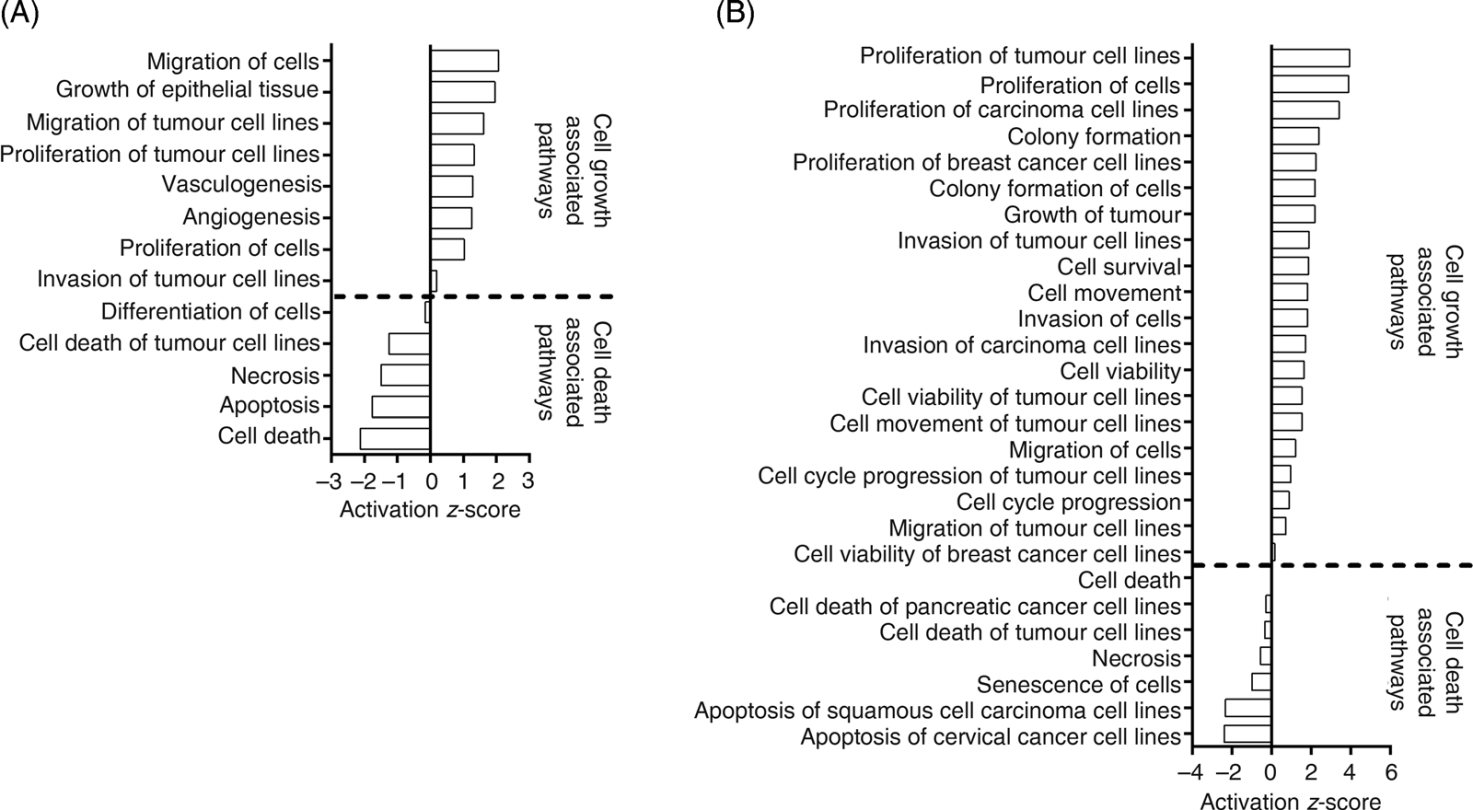
Pathway analysis of the effect of folic acid treatment on (A) MCF10A and (B) Hs578T. Pathway analysis was performed using Ingenuity Pathway Analysis (IPA). Activation *z*-scores show the predicted activation of each pathway based on the altered transcripts in each cell line. All activation *z*-scores have *P* < 0·05.

In MCF10A cells, FA treatment increased the predicted activation scores in ‘migration of cells’, ‘growth of epithelial tissue’, ‘proliferation of tumour cell lines’, ‘vasculogenesis’ and ‘angiogenesis’ pathways. FA treatment decreased the predicted activation scores for ‘apoptosis’ and ‘cell death’ pathways (Fig. 2(A)).

In Hs578T cells, FA treatment increased the predicted activation scores of ‘proliferation of tumour cell lines’, ‘cell survival’, ‘invasion of cells’ and ‘cell cycle progression of tumour cell lines’ pathways (Fig. 2(B)). FA treatment decreased the predicted activation scores of ‘Cell Death’ and ‘Senescence of Cells’ pathways (Fig. 2(B)).

### Upstream regulators are predicted to alter expression in Hs578T cells

**Fig. 3.**
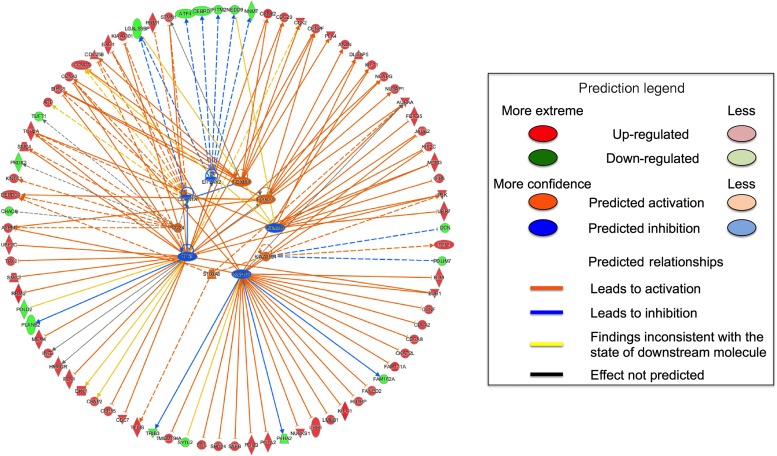
Firework plot of predicted upstream regulators in Hs578T cells. Pathway analysis was performed using Ingenuity Pathway Analysis (IPA). Upstream regulators with predicted activation are shown in orange and those with predicted inhibition are shown in blue. Down-regulated transcripts are shown in blue, up-regulated transcripts are shown in red. The relationships between the altered transcripts and the predicted upstream regulators are indicated by orange lines for activation, blue lines for inhibition and yellow lines for inconsistent results. Dotted lines indicate theoretical pathway relationships.

Only the Hs578T data set contained enough altered transcripts to conduct analysis of upstream regulators. Ten upstream regulators were predicted based on the changes in gene expression induced by FA. The activities of *FOXM1*, *FOXO1*, *CD24*, *KIAA1524* and *S100A6* were predicted to be increased, and the activities of *NUPR1*, *TP53*, *EIF2AK2*, *CDKN1A* and *KDM5B* were predicted to be decreased (Fig. 3). These predicted upstream regulators were identified based on the changes in expression of transcripts regulated by these proteins.

### Breast cancer cell lines exhibit differential expression of folate transporters

**Fig. 4.**
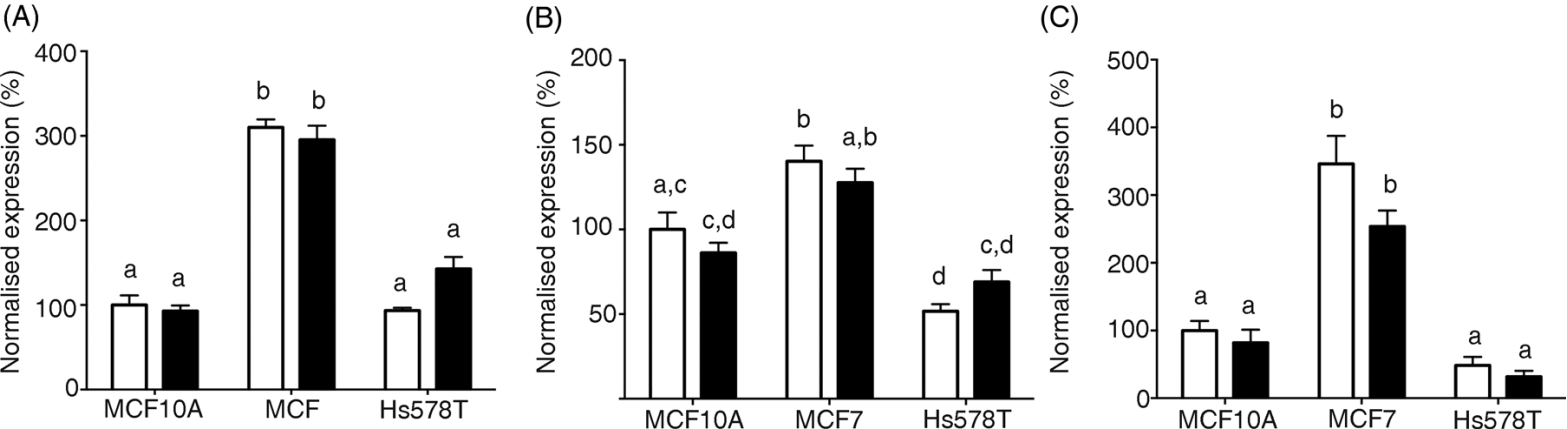
Expression of folate receptors in breast cancer cell lines. The expression of (A) *SLC19A1*, (B) *SLC46A1* and (C) *FOLR1* were measured in MCF10A, MCF7 and Hs578T treated with folic acid (100 nmol/l; ■) using quantitative RT-PCR. Values are means, with standard errors represented by vertical bars. Expression levels were normalised to glyceraldehyde 3-phosphate dehydrogenase (*GAPDH*) and are relative to MCF10A cells treated with 0 nmol/l folic acid (□). Data were analysed by two-way ANOVA using Bonferroni's *post hoc* correction. Mean values with unlike letters were significantly different (*P* < 0·05).

The expression of *SLC19A1* (RFCP) and *SLC46A1* (PCFT), which are involved in the uptake of unmetabolised FA^(^^22^^)^, and *FOLR1* (FRα)^(^^26^^)^, which has been linked to BC outcomes, were measured in MCF10A, MCF7 and Hs578T cell lines. In both the control and treated cells, expression of each folate transporter was significantly higher in MCF7 cells compared with both MCF10A and Hs578T cells (Fig. 4). There were no differences in expression of *SLC19A1* or *FOLR1* between the MCF10A and Hs578T cell lines, in either the control or treated cells (Fig. 4(A), (C)). Expression of *SLC46A1* was significantly lower in Hs578T cells compared with MCF10A cells (Fig. 4(B)). However, this effect was lost after FA treatment (Fig. 4(B)).

## Discussion

FA treatment induced cell type-related changes in gene expression such that the number of transcripts that were expressed differentially and the proportions of up- and down-regulated transcripts differed between cell lines. The proportion of transcripts that showed altered expression in only one cell line in response to FA treatment were MCF10A (89 %), MCF7 (75 %) and Hs578T (97 %). Only the expression of the cancer-associated gene *TAGLN*, which encodes transgelin, was altered by FA in all three cell lines. However, this transcript was down-regulated in MCF10A and Hs578T cells, and up-regulated in MCF7 cells. *TAGLN* has been shown previously to be down-regulated in breast, colon and prostate cancers and in virally transformed cells^(^^34^^,^^35^^)^, and hence has been proposed as an early marker of transformation and tumour progression^(^^34^^)^. Conversely, over-expression of *TAGLN* has been associated with suppression of cell proliferation and invasion, and promotion of apoptosis in colorectal carcinoma^(^^36^^)^. Thus the changes in *TAGLN* expression induced by FA treatment would be consistent with promotion of a cancer phenotype in MCF10A and Hs578T cells, but suppression of a cancer phenotype in MCF7 cells. Together these findings support the suggestion that the effect of FA on gene transcription is dependent upon the phenotype of the cells such that cells of similar histological origin, specifically adenocarcinomas, responded differentially.

Too few genes exhibited altered expression in response to FA in MCF7 cells to allow pathway analysis. However, both the non-transformed MCF10A cells and the adenoma-derived Hs578T cells showed up-regulation of pathways associated with tumorigenesis including cell migration, cell proliferation and vascularisation, while pathways associated with apoptosis and cell differentiation were down-regulated. These findings suggest that FA tended to induce changes across the transcriptome that were consistent with the effect on TAGLN and which would tend to promote a cancer phenotype.

Too few genes in MCF10A or MCF7 cells showed altered expression in response to FA for analysis of upstream regulators. However, ten upstream regulators were identified for the Hs578T cells. *FOXM1*, *FOXO1*, *CD24*, *KIAA1524* and S100A6 were predicted to have increased activity, and *KDM5B*, *CDKN1A*, *EIF2AK2*, *TP53* and *NUPR1* were predicted to have decreased activity. Some of these genes have functions that relate to BC development and progression including *FOXM*^(^^37^^)^, *FOXO1*^(^^38^^)^, *KIAA1524*^(^^39^^)^ and *S100A6*^(^^40^^)^. Furthermore, *NUPR1* is a mediator of metastatic growth that participates in early stages, but not late stages, of BC development^(^^41^^)^. This suggests that these cells develop a less stable carcinoma phenotype in response to FA. *CDKN1A* and *TP53* have been shown to function as tumour-suppressor genes^(^^42^^,^^43^^)^ and hence FA induced decreased capacity for cell death. Thus consistent with the pathway analysis, assessment of upstream regulators suggests that FA induced increased potential for cell proliferation and a more aggressive cancer phenotype.

It is not possible to deduce from these analyses the precise mechanism by which FA treatment led to induction of differential changes in the expression of the transcriptome of these cell lines. However, one possible mechanism is differences in the uptake of FA leading to different intracellular FA concentrations. It has been reported previously that two folate transporters are associated with cancer outcome^(^^23^^–^^25^^,^^44^^,^^45^^)^. The present findings show that *SLC19A1*, *SLC46A1* and *FOLR1* are differentially expressed between the three cell lines investigated. *SLC19A1* and *FOLR1* expression was approximately three-fold greater in MCF7 cells compared with MCF10A or Hs578T cells. *SLC46A1* expression was similar in MCF10A and MCF7cells, which was approximately two-fold greater than in Hs578T cells. However, the expression of these folate transporters did not relate in a simple manner to the number of transcripts that showed altered expression in response to FA. The effect of differential expression of each folate transporter on FA uptake cannot be deduced from these data and the effect of any differences in FA uptake on the BC transcriptome may be modified by the capacity of these cell lines to metabolise FA.

The major limitations of the present study are that established cell lines were used, which exhibit many differences other than hormone receptor status that could confound the results. In order to gain a better understanding of clinical BC, primary cells derived from tumours of different hormone status and healthy cells from the same individual would provide more robust findings. Although the differences in gene expression levels were relatively small, the pathway analysis shows that the findings are consistent. However, the impact of differences in gene expression on cell function remains to be determined. Furthermore, the cell lines were treated with FA for a relatively short period of time compared with the exposure of BC cells in women who take dietary supplements that contain FA. In addition, one previous study has shown that BC cell lines undergo phenotypic changes over time and that such changes are modified by whether the cells were grown as a monolayer or a three-dimensional structure^(^^46^^)^. Such effects of duration and type of culture system may have influenced the results of the present study. Nevertheless, the present findings provide insights into the differential effects of FA on BC and proof-of-concept evidence of possible mechanism of FA action that could form the basis for studies in more physiological systems.

These findings provide for the first time proof-of-concept evidence that exposure to FA at a concentration which can be achieved in humans taking FA dietary supplements^(^^28^^)^ can induce cell type-specific changes in the transcriptome of normal and transformed human breast cells. Such effects may be greater in populations exposed to a diet fortified with FA^(^^47^^,^^48^^)^. Furthermore, these results suggest that there may be differences in the effect of FA on BC subtypes. If these findings were replicated in primary tumour tissue from women who take FA supplements, then they would have important implications for dietary recommendations to women with BC and for the general population. However, the extent to which generalised recommendations could be made may be limited by the cell phenotype-specific nature of the effects of FA on gene regulation. However, it may be possible to tailor recommendations to individual patients to reduce the progress of BC.

## Supplementary material

For supplementary material accompanying this paper visit http://dx.doi.org/10.1017/jns.2016.8. click here to view supplementary material

## Supplementary material

The supplementary material for this article can be found at http://dx.doi.org/10.1017/jns.2016.8
