# Supplementary material for: Folic acid induces cell type-specific changes in the transcriptome of breast cancer cell lines: a proof-of-concept study
Source: J Nutr Sci. 2016 Apr 26;5:e17. doi: 10.1017/jns.2016.8 (PMC4891697; doi:10.1017/jns.2016.8)
Supplement: Supplementary file 1 [file S2048679016000082sup001.doc]

**Supplementary material**

**Folic acid induces cell type-specific changes in the transcriptome of breast cancer cell lines: a proof-of-concept study**

**Price, R.J., Lillycrop, K.A. and Burdge, G.C.**

**Supplementary Fig. S1.** Heat map of significantly altered transcripts from MCF10A, MCF7 and Hs578T cell lines. MeV software was used to visualise the significantly altered transcripts from each cell line alongside the corresponding transcripts in the other cell lines. Red indicates an increase in expression and green indicates a decrease.

**
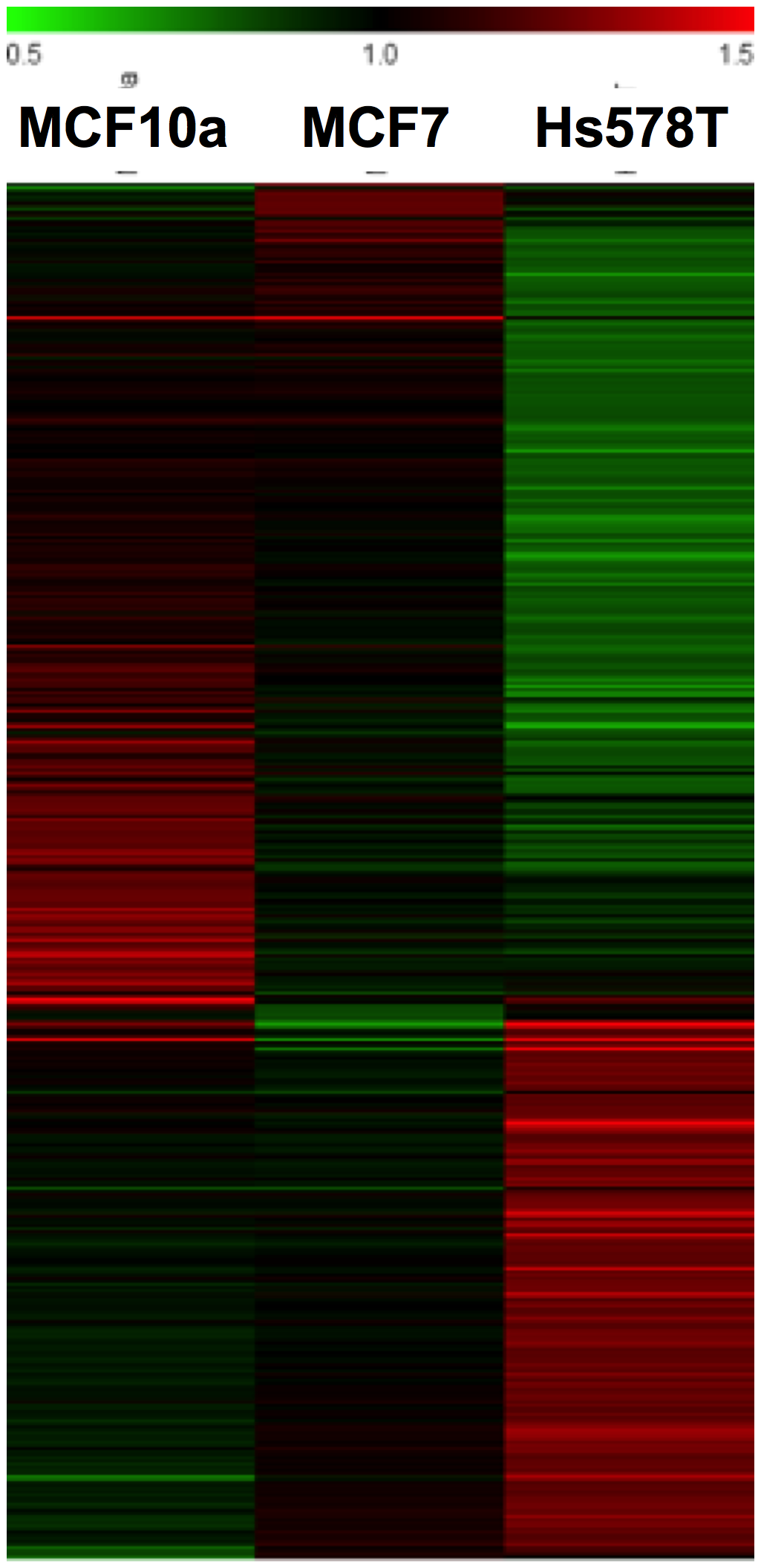
**

**Supplementary Table S1.** Transcripts showing significantly altered expression in MCF10A cells in response to FA treatment

| **Gene Symbol** | **NCBI Reference Sequence** | **Gene Description** | **Fold Change** | **P Value** |
| --- | --- | --- | --- | --- |
| MIR1978 | NR_031742.1 | Homo sapiens microRNA 1978 (MIR1978), microRNA. | -1.31 | 0.006 |
| OKL38 | NM_013370.2 | Homo sapiens pregnancy-induced growth inhibitor (OKL38), transcript variant 1, mRNA. | -1.30 | 0.005 |
| TAGLN | NM_003186.3 | Homo sapiens transgelin (TAGLN), transcript variant 2, mRNA. | -1.25 | 0.031 |
| CDC42BPA | NM_014826.3 | Homo sapiens CDC42 binding protein kinase alpha (DMPK-like) (CDC42BPA), transcript variant B, mRNA. | -1.20 | 0.003 |
| CNOT1 | NM_016284.3 | Homo sapiens CCR4-NOT transcription complex, subunit 1 (CNOT1), transcript variant 1, mRNA. | -1.20 | 0.009 |
| EI24 | NM_004879.3 | Homo sapiens etoposide induced 2.4 mRNA (EI24), transcript variant 1, mRNA. | 1.20 | 0.022 |
| HNRPC | NM_031314.1 | Homo sapiens heterogeneous nuclear ribonucleoprotein C (C1/C2) (HNRPC), transcript variant 1, mRNA. | 1.20 | 0.045 |
| ID2 | NM_002166.4 | Homo sapiens inhibitor of DNA binding 2, dominant negative helix-loop-helix protein (ID2), mRNA. | 1.20 | 0.004 |
| LOC441743 | NM_001045548.1 | Homo sapiens similar to C367G8.3 (novel protein similar to RPL23A (60S ribosomal protein L23A)) (LOC441743), mRNA. | 1.20 | 0.015 |
| IL8 | NM_000584.2 | Homo sapiens interleukin 8 (IL8), mRNA. | 1.20 | 0.026 |
| NME2 | NM_001018138.1 | Homo sapiens non-metastatic cells 2, protein (NM23B) expressed in (NME2), transcript variant 3, mRNA. | 1.20 | 0.000 |
| VAMP4 | NM_003762.2 | Homo sapiens vesicle-associated membrane protein 4 (VAMP4), mRNA. | 1.20 | 0.012 |
| NUP62 | NM_153719.2 | Homo sapiens nucleoporin 62kDa (NUP62), transcript variant 1, mRNA. | 1.20 | 0.023 |
| IGFBP6 | NM_002178.2 | Homo sapiens insulin-like growth factor binding protein 6 (IGFBP6), mRNA. | 1.20 | 0.020 |
| SNURF | NM_003097.3 | Homo sapiens SNRPN upstream reading frame (SNURF), transcript variant 1, mRNA. | 1.20 | 0.025 |
| LOC100129685 | XM_001723814.1 | PREDICTED: Homo sapiens hypothetical protein LOC100129685 (LOC100129685), mRNA. | 1.21 | 0.002 |
| HMGN2 | NM_005517.2 | Homo sapiens high-mobility group nucleosomal binding domain 2 (HMGN2), mRNA. | 1.21 | 0.034 |
| LOC644131 | XM_937322.1 | PREDICTED: Homo sapiens similar to chaperonin containing TCP1, subunit 8 (theta) (LOC644131), mRNA. | 1.21 | 0.016 |
| SNX5 | NM_152227.1 | Homo sapiens sorting nexin 5 (SNX5), transcript variant 1, mRNA. | 1.21 | 0.047 |
| LOC389787 | XM_497072.2 | PREDICTED: Homo sapiens similar to Translationally-controlled tumor protein (TCTP) (p23) (Histamine-releasing factor) (HRF) (Fortilin) (LOC389787), mRNA. | 1.22 | 0.043 |
| LOC645979 | XM_001721589.1 | PREDICTED: Homo sapiens similar to ribosomal protein S26 (LOC645979), mRNA. | 1.22 | 0.023 |
| TWIST2 | XM_941980.1 | Homo sapiens twist homolog 2 (Drosophila) (TWIST2), mRNA. | 1.22 | 0.014 |
|  | Hs.543887 | AGENCOURT_14535501 NIH_MGC_191 Homo sapiens cDNA clone IMAGE:30415823 5, mRNA sequence | 1.22 | 0.007 |
| CXCL1 | NM_001511.1 | Homo sapiens chemokine (C-X-C motif) ligand 1 (melanoma growth stimulating activity, alpha) (CXCL1), mRNA. | 1.22 | 0.050 |
| LOC441073 | XR_018937.2 | PREDICTED: Homo sapiens misc_RNA (LOC441073), miscRNA. | 1.22 | 0.018 |
| PTGES3 | NM_006601.4 | Homo sapiens prostaglandin E synthase 3 (cytosolic) (PTGES3), mRNA. | 1.22 | 0.012 |
| LOC728253 | XM_001128502.2 | PREDICTED: Homo sapiens hypothetical LOC728253 (LOC728253), mRNA. | 1.22 | 0.049 |
| LOC729362 | XR_041521.1 | PREDICTED: Homo sapiens misc_RNA (LOC729362), miscRNA. | 1.22 | 0.039 |
| LOC729340 | XR_039650.1 | PREDICTED: Homo sapiens misc_RNA (LOC729340), miscRNA. | 1.22 | 0.019 |
| LOC100130835 | XR_038851.1 | PREDICTED: Homo sapiens misc_RNA (LOC100130835), miscRNA. | 1.22 | 0.010 |
| LOC100130070 | XM_001723889.1 | PREDICTED: Homo sapiens similar to metallopanstimulin (LOC100130070), mRNA. | 1.23 | 0.033 |
| LOC100131526 | XR_038945.1 | PREDICTED: Homo sapiens misc_RNA (LOC100131526), miscRNA. | 1.23 | 0.027 |
| LOC347292 | XM_294581.2 | PREDICTED: Homo sapiens similar to ribosomal protein L36 (LOC347292), mRNA. | 1.23 | 0.002 |
| LOC727984 | XR_015259.2 | PREDICTED: Homo sapiens misc_RNA (LOC727984), miscRNA. | 1.23 | 0.003 |
| SFRS13A | NM_054016.1 | Homo sapiens splicing factor, arginine/serine-rich 13A (SFRS13A), transcript variant 2, mRNA. | 1.23 | 0.005 |
| LOC100131971 | XM_001720161.1 | PREDICTED: Homo sapiens similar to 40S ribosomal protein S26 (LOC100131971), mRNA. | 1.23 | 0.025 |
| LOC100134504 | XM_001725687.1 | PREDICTED: Homo sapiens hypothetical protein LOC100134504 (LOC100134504), mRNA. | 1.23 | 0.018 |
| LOC441377 | XM_938599.1 | PREDICTED: Homo sapiens similar to 40S ribosomal protein S26 (LOC441377), mRNA. | 1.23 | 0.049 |
| LOC644790 | XM_927887.3 | PREDICTED: Homo sapiens hypothetical LOC644790 (LOC644790), mRNA. | 1.23 | 0.008 |
| LOC388076 | XM_001722259.1 | PREDICTED: Homo sapiens hypothetical LOC388076 (LOC388076), mRNA. | 1.24 | 0.019 |
| TDP1 | NM_018319.3 | Homo sapiens tyrosyl-DNA phosphodiesterase 1 (TDP1), transcript variant 1, mRNA. | 1.24 | 0.017 |
| LOC100129599 | XM_001724539.1 | PREDICTED: Homo sapiens similar to mCG7602 (LOC100129599), mRNA. | 1.24 | 0.018 |
| YWHAG | NM_012479.2 | Homo sapiens tyrosine 3-monooxygenase/tryptophan 5-monooxygenase activation protein, gamma polypeptide (YWHAG), mRNA. | 1.24 | 0.034 |
| TCP1 | NM_030752.2 | Homo sapiens t-complex 1 (TCP1), transcript variant 1, mRNA. | 1.24 | 0.011 |
| SAV1 | NM_021818.2 | Homo sapiens salvador homolog 1 (Drosophila) (SAV1), mRNA. | 1.25 | 0.034 |
| LOC100131672 | XR_039068.1 | PREDICTED: Homo sapiens misc_RNA (LOC100131672), miscRNA. | 1.25 | 0.032 |
| RNF13 | NM_183381.1 | Homo sapiens ring finger protein 13 (RNF13), transcript variant 4, mRNA. | 1.25 | 0.042 |
| C10orf32 | NM_144591.1 | Homo sapiens chromosome 10 open reading frame 32 (C10orf32), mRNA. | 1.25 | 0.023 |
| C1orf63 | NM_020317.3 | Homo sapiens chromosome 1 open reading frame 63 (C1orf63), mRNA. | 1.26 | 0.001 |
| LOC100130154 | XM_001717333.1 | PREDICTED: Homo sapiens similar to thymosin, beta 10 (LOC100130154), mRNA. | 1.26 | 0.005 |
| RPL7 | NM_000971.3 | Homo sapiens ribosomal protein L7 (RPL7), mRNA. | 1.26 | 0.013 |
| LOC729208 | XR_039643.1 | PREDICTED: Homo sapiens misc_RNA (LOC729208), miscRNA. | 1.27 | 0.009 |
| FTHL3 | NR_002201.1 | Homo sapiens ferritin, heavy polypeptide-like 3 (FTHL3), non-coding RNA. | 1.27 | 0.015 |
| RPL10A | NM_007104.4 | Homo sapiens ribosomal protein L10a (RPL10A), mRNA. | 1.27 | 0.032 |
| LOC100132547 | XR_039468.1 | PREDICTED: Homo sapiens misc_RNA (LOC100132547), miscRNA. | 1.28 | 0.020 |
| DCN | NM_133503.2 | Homo sapiens decorin (DCN), transcript variant A2, mRNA. | 1.29 | 0.049 |
| LOC727821 | XR_037166.1 | PREDICTED: Homo sapiens misc_RNA (LOC727821), miscRNA. | 1.29 | 0.006 |
| PLD1 | NM_002662.2 | Homo sapiens phospholipase D1, phosphatidylcholine-specific (PLD1), mRNA. | 1.30 | 0.014 |
| LOC100131572 | XM_001725183.1 | PREDICTED: Homo sapiens similar to hCG1783679 (LOC100131572), mRNA. | 1.30 | 0.024 |
| LOC389156 | XR_017589.2 | PREDICTED: Homo sapiens misc_RNA (LOC389156), miscRNA. | 1.30 | 0.021 |
| LOC645236 | XM_928275.1 | PREDICTED: Homo sapiens similar to similar to RPL23AP7 protein (LOC645236), mRNA. | 1.33 | 0.028 |
| LOC644914 | XM_930111.2 | PREDICTED: Homo sapiens similar to H3 histone, family 3B (LOC644914), mRNA. | 1.33 | 0.011 |
| LOC643031 | XM_926402.1 | PREDICTED: Homo sapiens similar to NADH dehydrogenase subunit 5 (LOC643031), mRNA. | 1.34 | 0.025 |
| LOC128192 | XM_001725191.1 | PREDICTED: Homo sapiens hypothetical LOC128192 (LOC128192), mRNA. | 1.34 | 0.026 |
| LOC347376 | XM_937928.1 | PREDICTED: Homo sapiens similar to H3 histone, family 3B (LOC347376), mRNA. | 1.34 | 0.006 |
| LOC100131905 | XR_039334.1 | PREDICTED: Homo sapiens misc_RNA (LOC100131905), miscRNA. | 1.35 | 0.034 |
| LOC440063 | XR_018394.2 | PREDICTED: Homo sapiens misc_RNA (LOC440063), miscRNA. | 1.36 | 0.023 |
| RPLP1 | NM_001003.2 | Homo sapiens ribosomal protein, large, P1 (RPLP1), transcript variant 1, mRNA. | 1.37 | 0.042 |
| LOC645691 | XM_928701.3 | PREDICTED: Homo sapiens similar to heterogeneous nuclear ribonucleoprotein A1 (LOC645691), mRNA. | 1.38 | 0.012 |
| LOC441019 | XM_498969.2 | PREDICTED: Homo sapiens hypothetical LOC441019 (LOC441019), mRNA. | 1.40 | 0.006 |
| LOC653737 | XM_929340.3 | PREDICTED: Homo sapiens hypothetical LOC653737 (LOC653737), mRNA. | 1.41 | 0.028 |
| RPL12P6 | XR_016704.2 | PREDICTED: Homo sapiens misc_RNA (RPL12P6), miscRNA. | 1.41 | 0.017 |
| LOC653156 | XM_001718864.1 | PREDICTED: Homo sapiens similar to hCG1782414 (LOC653156), mRNA. | 1.42 | 0.032 |
| HSPE1 | NM_002157.1 | Homo sapiens heat shock 10kDa protein 1 (chaperonin 10) (HSPE1), mRNA. | 1.46 | 0.020 |
| CLEC2D | NM_013269.2 | Homo sapiens C-type lectin domain family 2, member D (CLEC2D), transcript variant 1, mRNA. | 1.62 | 0.035 |

**Supplementary Table S2.** Transcripts showing significantly altered expression in MCF7 cells in response to FA treatment

| **Gene Symbol** | **NCBI Reference Sequence** | **Gene Description** | **Fold Change** | **P Value** |
| --- | --- | --- | --- | --- |
| LOC100132394 | XM_001713809.1 | PREDICTED: Homo sapiens hypothetical protein LOC100132394 (LOC100132394), mRNA. | -1.48 | 0.034 |
| LOC100134364 | XM_001713810.1 | PREDICTED: Homo sapiens hypothetical protein LOC100134364 (LOC100134364), mRNA. | -1.43 | 0.021 |
| NBPF8 | XM_001726946.1 | PREDICTED: Homo sapiens neuroblastoma breakpoint family, member 8 (NBPF8), mRNA. | -1.23 | 0.015 |
| AFF4 | NM_014423.2 | Homo sapiens AF4/FMR2 family, member 4 (AFF4), mRNA. | -1.23 | 0.050 |
| LYPD1 | NM_144586.3 | Homo sapiens LY6/PLAUR domain containing 1 (LYPD1), transcript variant 1, mRNA. | -1.22 | 0.004 |
| C3orf57 | NM_145035.2 | Homo sapiens chromosome 3 open reading frame 57 (C3orf57), mRNA. | -1.21 | 0.015 |
| PERP | NM_022121.2 | Homo sapiens PERP, TP53 apoptosis effector (PERP), mRNA. | -1.21 | 0.032 |
| CLCN7 | NM_001287.3 | Homo sapiens chloride channel 7 (CLCN7), mRNA. | -1.20 | 0.024 |
| PCDHA1 | NM_018907.2 | Homo sapiens protocadherin alpha 1 (PCDHA1), transcript variant 3, mRNA. | -1.20 | 0.002 |
| HINT3 | NM_138571.4 | Homo sapiens histidine triad nucleotide binding protein 3 (HINT3), mRNA. | -1.20 | 0.018 |
| COMMD5 | NM_014066.3 | Homo sapiens COMM domain containing 5 (COMMD5), transcript variant 1, mRNA. | 1.20 | 0.045 |
| MRPL21 | NM_181514.1 | Homo sapiens mitochondrial ribosomal protein L21 (MRPL21), nuclear gene encoding mitochondrial protein, transcript variant 4, mRNA. | 1.20 | 0.003 |
| SNORD17 | NR_003045.1 | Homo sapiens small nucleolar RNA, C/D box 17 (SNORD17), small nucleolar RNA. | 1.20 | 0.004 |
|  | Hs.473191 | Homo sapiens cDNA FLJ34428 fis, clone HLUNG2000761 | 1.21 | 0.018 |
| TAGLN | NM_003186.3 | Homo sapiens transgelin (TAGLN), transcript variant 2, mRNA. | 1.21 | 0.001 |
| KRT86 | NM_002284.3 | Homo sapiens keratin 86 (KRT86), mRNA. | 1.21 | 0.001 |
| LOC147727 | NR_024333.1 | Homo sapiens hypothetical LOC147727 (LOC147727), non-coding RNA. | 1.22 | 0.000 |
| C15orf44 | XM_940546.1 | Homo sapiens chromosome 15 open reading frame 44 (C15orf44), transcript variant 2, mRNA. | 1.22 | 0.007 |
| BCAS1 | NM_003657.1 | Homo sapiens breast carcinoma amplified sequence 1 (BCAS1), mRNA. | 1.22 | 0.014 |
| THEM4 | NM_053055.3 | Homo sapiens thioesterase superfamily member 4 (THEM4), mRNA. | 1.23 | 0.001 |
| RN5S9 | NR_023371.1 | Homo sapiens RNA, 5S ribosomal 9 (RN5S9), ribosomal RNA. | 1.24 | 0.003 |
| GABBR2 | NM_005458.5 | Homo sapiens gamma-aminobutyric acid (GABA) B receptor, 2 (GABBR2), mRNA. | 1.24 | 0.002 |
| RPL8 | NM_033301.1 | Homo sapiens ribosomal protein L8 (RPL8), transcript variant 2, mRNA. | 1.29 | 0.001 |
| HSPE1 | NM_002157.1 | Homo sapiens heat shock 10kDa protein 1 (chaperonin 10) (HSPE1), mRNA. | 1.54 | 0.026 |

**Supplementary Table S3.** Transcripts showing significantly altered expression in Hs578T cells in response to FA treatment

| **Gene Symbol** | **NCBI Reference Sequence** | **Gene Description** | **Fold Change** | **P Value** |
| --- | --- | --- | --- | --- |
| LOC728188 | XM_001126103.2 | PREDICTED: Homo sapiens similar to phosphoglycerate mutase processed protein (LOC728188), mRNA. | -1.50 | 0.002 |
| MXRA8 | NM_032348.2 | Homo sapiens matrix-remodelling associated 8 (MXRA8), mRNA. | -1.50 | 0.006 |
| LOC148430 | XR_038750.1 | PREDICTED: Homo sapiens misc_RNA (LOC148430), miscRNA. | -1.49 | 0.016 |
| KRT17P3 | XR_015626.2 | PREDICTED: Homo sapiens misc_RNA (KRT17P3), miscRNA. | -1.48 | 0.000 |
| LOC729009 | XR_042330.1 | PREDICTED: Homo sapiens misc_RNA (LOC729009), miscRNA. | -1.47 | 0.022 |
| LOC729926 | XR_038426.1 | PREDICTED: Homo sapiens misc_RNA (LOC729926), miscRNA. | -1.47 | 0.001 |
| PGAM1 | NM_002629.2 | Homo sapiens phosphoglycerate mutase 1 (brain) (PGAM1), mRNA. | -1.45 | 0.005 |
| C5orf46 | NM_206966.1 | Homo sapiens chromosome 5 open reading frame 46 (C5orf46), mRNA. | -1.44 | 0.000 |
| C1R | NM_001733.4 | Homo sapiens complement component 1, r subcomponent (C1R), mRNA. | -1.44 | 0.001 |
| LOC440991 | XR_017665.2 | PREDICTED: Homo sapiens misc_RNA (LOC440991), miscRNA. | -1.39 | 0.018 |
| KRT16 | NM_005557.2 | Homo sapiens keratin 16 (focal non-epidermolytic palmoplantar keratoderma) (KRT16), mRNA. | -1.39 | 0.003 |
| MAEA | NM_005882.3 | Homo sapiens macrophage erythroblast attacher (MAEA), transcript variant 2, mRNA. | -1.38 | 0.001 |
| ACTG1 | NM_001614.2 | Homo sapiens actin, gamma 1 (ACTG1), mRNA. | -1.37 | 0.003 |
| FBLN1 | NM_001996.2 | Homo sapiens fibulin 1 (FBLN1), transcript variant A, mRNA. | -1.37 | 0.000 |
| IL32 | NM_001012633.1 | Homo sapiens interleukin 32 (IL32), transcript variant 4, mRNA. | -1.36 | 0.003 |
| PGAM4 | XM_926168.1 | Homo sapiens phosphoglycerate mutase family member 4 (PGAM4), mRNA. | -1.36 | 0.004 |
| LOC730029 | XM_001724847.1 | PREDICTED: Homo sapiens similar to hCG1997137, transcript variant 2 (LOC730029), mRNA. | -1.36 | 0.026 |
| RPL8 | NM_033301.1 | Homo sapiens ribosomal protein L8 (RPL8), transcript variant 2, mRNA. | -1.35 | 0.000 |
| WDR1 | NM_017491.3 | Homo sapiens WD repeat domain 1 (WDR1), transcript variant 1, mRNA. | -1.35 | 0.007 |
| TES | NM_015641.2 | Homo sapiens testis derived transcript (3 LIM domains) (TES), transcript variant 1, mRNA. | -1.34 | 0.000 |
| LOC100128892 | XM_001716912.1 | PREDICTED: Homo sapiens hypothetical protein LOC100128892 (LOC100128892), mRNA. | -1.34 | 0.010 |
| LOC644774 | XM_927868.1 | PREDICTED: Homo sapiens similar to Phosphoglycerate kinase 1 (LOC644774), mRNA. | -1.34 | 0.007 |
| HCG4 | XR_001115.1 | Homo sapiens HLA complex group 4 (HCG4), non-coding RNA. | -1.33 | 0.000 |
| ASAM | NM_024769.2 | Homo sapiens adipocyte-specific adhesion molecule (ASAM), mRNA. | -1.33 | 0.015 |
| CCDC80 | NM_199511.1 | Homo sapiens coiled-coil domain containing 80 (CCDC80), transcript variant 1, mRNA. | -1.33 | 0.008 |
| LOC730996 | XM_943879.1 | PREDICTED: Homo sapiens similar to chromosome 1 open reading frame 80 (LOC730996), mRNA. | -1.32 | 0.030 |
| LOC732007 | XM_940732.1 | PREDICTED: Homo sapiens similar to Phosphoglycerate mutase 1 (Phosphoglycerate mutase isozyme B) (PGAM-B) (BPG-dependent PGAM 1) (LOC732007), mRNA. | -1.32 | 0.009 |
| POTEE | NM_001083538.1 | Homo sapiens POTE ankyrin domain family, member E (POTEE), mRNA. | -1.32 | 0.008 |
| SNRPN | NM_005678.3 | Homo sapiens small nuclear ribonucleoprotein polypeptide N (SNRPN), transcript variant 3, mRNA. | -1.31 | 0.003 |
| LOC128192 | XM_001725191.1 | PREDICTED: Homo sapiens hypothetical LOC128192 (LOC128192), mRNA. | -1.30 | 0.048 |
| LOC441506 | XR_017565.2 | PREDICTED: Homo sapiens misc_RNA (LOC441506), miscRNA. | -1.30 | 0.005 |
| NIPSNAP1 | NM_003634.1 | Homo sapiens nipsnap homolog 1 (C. elegans) (NIPSNAP1), mRNA. | -1.30 | 0.000 |
| LOC643997 | XM_292963.5 | PREDICTED: Homo sapiens similar to peptidylprolyl isomerase A isoform 1 (LOC643997), mRNA. | -1.29 | 0.012 |
| LOC100132673 | XR_039018.1 | PREDICTED: Homo sapiens misc_RNA (LOC100132673), miscRNA. | -1.29 | 0.018 |
| ALDOA | NM_184041.1 | Homo sapiens aldolase A, fructose-bisphosphate (ALDOA), transcript variant 2, mRNA. | -1.29 | 0.010 |
| LOC100132535 | XR_038625.1 | PREDICTED: Homo sapiens misc_RNA (LOC100132535), miscRNA. | -1.29 | 0.003 |
| LGALS3BP | NM_005567.2 | Homo sapiens lectin, galactoside-binding, soluble, 3 binding protein (LGALS3BP), mRNA. | -1.29 | 0.002 |
| NGRN | NM_016645.2 | Homo sapiens neugrin, neurite outgrowth associated (NGRN), transcript variant 1, mRNA. | -1.29 | 0.003 |
| MSC | NM_005098.2 | Homo sapiens musculin (activated B-cell factor-1) (MSC), mRNA. | -1.29 | 0.000 |
| LOC728139 | XR_015301.2 | PREDICTED: Homo sapiens misc_RNA (LOC728139), miscRNA. | -1.29 | 0.001 |
| ACO2 | NM_001098.2 | Homo sapiens aconitase 2, mitochondrial (ACO2), nuclear gene encoding mitochondrial protein, mRNA. | -1.29 | 0.000 |
| LOC643300 | XM_931981.1 | PREDICTED: Homo sapiens similar to 60 kDa heat shock protein, mitochondrial precursor (Hsp60) (60 kDa chaperonin) (CPN60) (Heat shock protein 60) (HSP-60) (Mitochondrial matrix protein P1) (P60 lymphocyte protein) (HuCHA60) (LOC643300), mRNA. | -1.29 | 0.017 |
|  | Hs.579631 | AGENCOURT_10229596 NIH_MGC_141 Homo sapiens cDNA clone IMAGE:6563923 5, mRNA sequence | -1.28 | 0.008 |
| LDOC1 | NM_012317.2 | Homo sapiens leucine zipper, down-regulated in cancer 1 (LDOC1), mRNA. | -1.28 | 0.020 |
| FTHL3 | NR_002201.1 | Homo sapiens ferritin, heavy polypeptide-like 3 (FTHL3), non-coding RNA. | -1.28 | 0.045 |
| LOC645979 | XM_001721589.1 | PREDICTED: Homo sapiens similar to ribosomal protein S26 (LOC645979), mRNA. | -1.28 | 0.047 |
| LOC729779 | XR_019592.2 | PREDICTED: Homo sapiens misc_RNA (LOC729779), miscRNA. | -1.28 | 0.006 |
| INHBE | NM_031479.3 | Homo sapiens inhibin, beta E (INHBE), mRNA. | -1.28 | 0.001 |
| CD151 | NM_004357.3 | Homo sapiens CD151 molecule (Raph blood group) (CD151), transcript variant 4, mRNA. | -1.28 | 0.011 |
| LOC402251 | XM_377933.3 | PREDICTED: Homo sapiens similar to eukaryotic translation elongation factor 1 alpha 2 (LOC402251), mRNA. | -1.28 | 0.032 |
| IDH1 | NM_005896.2 | Homo sapiens isocitrate dehydrogenase 1 (NADP+), soluble (IDH1), mRNA. | -1.28 | 0.031 |
| P704P | XM_926231.1 | PREDICTED: Homo sapiens prostate-specific P704P (P704P), mRNA. | -1.27 | 0.028 |
| HSPB6 | NM_144617.1 | Homo sapiens heat shock protein, alpha-crystallin-related, B6 (HSPB6), mRNA. | -1.27 | 0.017 |
| LOC650517 | XM_496202.2 | PREDICTED: Homo sapiens hypothetical LOC650517 (LOC650517), mRNA. | -1.27 | 0.001 |
| ANKRD37 | NM_181726.1 | Homo sapiens ankyrin repeat domain 37 (ANKRD37), mRNA. | -1.27 | 0.001 |
| G6PD | NM_000402.2 | Homo sapiens glucose-6-phosphate dehydrogenase (G6PD), transcript variant 1, mRNA. | -1.27 | 0.001 |
| KRT17 | NM_000422.1 | Homo sapiens keratin 17 (KRT17), mRNA. | -1.27 | 0.000 |
| COL6A2 | NM_058174.1 | Homo sapiens collagen, type VI, alpha 2 (COL6A2), transcript variant 2C2, mRNA. | -1.27 | 0.022 |
| EFEMP1 | NM_001039348.1 | Homo sapiens EGF-containing fibulin-like extracellular matrix protein 1 (EFEMP1), transcript variant 2, mRNA. | -1.27 | 0.001 |
| LOC651149 | XM_929125.1 | PREDICTED: Homo sapiens similar to 60S ribosomal protein L3 (L4) (LOC651149), mRNA. | -1.26 | 0.016 |
| COMT | NM_000754.2 | Homo sapiens catechol-O-methyltransferase (COMT), transcript variant MB-COMT, mRNA. | -1.26 | 0.024 |
| P4HA2 | NM_001017974.1 | Homo sapiens prolyl 4-hydroxylase, alpha polypeptide II (P4HA2), transcript variant 3, mRNA. | -1.26 | 0.010 |
| FTHL8 | NR_002203.1 | Homo sapiens ferritin, heavy polypeptide-like 8 (FTHL8) on chromosome X. | -1.26 | 0.021 |
| LOC341315 | XR_017616.1 | PREDICTED: Homo sapiens misc_RNA (LOC341315), miscRNA. | -1.26 | 0.002 |
| DCN | NM_001920.3 | Homo sapiens decorin (DCN), transcript variant A1, mRNA. | -1.26 | 0.037 |
| UBTD1 | NM_024954.3 | Homo sapiens ubiquitin domain containing 1 (UBTD1), mRNA. | -1.26 | 0.002 |
| TAGLN | NM_003186.3 | Homo sapiens transgelin (TAGLN), transcript variant 2, mRNA. | -1.26 | 0.006 |
| HERPUD1 | NM_001010990.1 | Homo sapiens homocysteine-inducible, endoplasmic reticulum stress-inducible, ubiquitin-like domain member 1 (HERPUD1), transcript variant 3, mRNA. | -1.26 | 0.002 |
| PI4KAP1 | XM_942088.1 | Homo sapiens phosphatidylinositol 4-kinase, catalytic, alpha pseudogene 1 (PI4KAP1), non-coding RNA. | -1.26 | 0.000 |
| TSPAN3 | NM_005724.4 | Homo sapiens tetraspanin 3 (TSPAN3), transcript variant 1, mRNA. | -1.25 | 0.028 |
| PRDX2 | NM_181738.1 | Homo sapiens peroxiredoxin 2 (PRDX2), nuclear gene encoding mitochondrial protein, transcript variant 3, mRNA. | -1.25 | 0.005 |
| SPG21 | NM_016630.3 | Homo sapiens spastic paraplegia 21 (autosomal recessive, Mast syndrome) (SPG21), transcript variant 1, mRNA. XM_945612 XM_945613 XM_945615 XM_945617 XM_945619 XM_945622 | -1.25 | 0.032 |
| C19orf60 | NM_001100418.1 | Homo sapiens chromosome 19 open reading frame 60 (C19orf60), transcript variant 1, mRNA. | -1.25 | 0.002 |
| KIAA1199 | NM_018689.1 | Homo sapiens KIAA1199 (KIAA1199), mRNA. | -1.25 | 0.002 |
| PLCG1 | NM_002660.2 | Homo sapiens phospholipase C, gamma 1 (PLCG1), transcript variant 2, mRNA. | -1.25 | 0.000 |
| PDLIM7 | NM_213636.1 | Homo sapiens PDZ and LIM domain 7 (enigma) (PDLIM7), transcript variant 1, mRNA. | -1.25 | 0.040 |
| LOC100134407 | XM_001715747.1 | PREDICTED: Homo sapiens hypothetical protein LOC100134407 (LOC100134407), mRNA. | -1.25 | 0.021 |
| XPOT | NM_007235.3 | Homo sapiens exportin, tRNA (nuclear export receptor for tRNAs) (XPOT), mRNA. | -1.24 | 0.045 |
| SLC29A4 | NM_153247.1 | Homo sapiens solute carrier family 29 (nucleoside transporters), member 4 (SLC29A4), mRNA. | -1.24 | 0.000 |
| ZMAT5 | NM_019103.2 | Homo sapiens zinc finger, matrin type 5 (ZMAT5), transcript variant 1, mRNA. | -1.24 | 0.002 |
| LOC100132060 | XM_001720144.1 | PREDICTED: Homo sapiens hypothetical protein LOC100132060 (LOC100132060), mRNA. | -1.24 | 0.030 |
| FTHL12 | NR_002205.1 | Homo sapiens ferritin, heavy polypeptide-like 12 (FTHL12) on chromosome 9. | -1.24 | 0.034 |
| PLAC9 | NM_001012973.1 | Homo sapiens placenta-specific 9 (PLAC9), mRNA. | -1.24 | 0.000 |
| CRELD1 | NM_001031717.1 | Homo sapiens cysteine-rich with EGF-like domains 1 (CRELD1), transcript variant 1, mRNA. | -1.24 | 0.002 |
| FAM127C | NM_001078173.1 | Homo sapiens family with sequence similarity 127, member C (FAM127C), mRNA. | -1.24 | 0.023 |
| LOC644029 | XM_944890.1 | PREDICTED: Homo sapiens similar to 60S ribosomal protein L7a (LOC644029), mRNA. | -1.24 | 0.004 |
| ZNF503 | NM_032772.3 | Homo sapiens zinc finger protein 503 (ZNF503), mRNA. | -1.24 | 0.005 |
| LOC285741 | XR_017420.2 | PREDICTED: Homo sapiens misc_RNA (LOC285741), miscRNA. | -1.24 | 0.015 |
| LOC727865 | XR_038568.1 | PREDICTED: Homo sapiens misc_RNA (LOC727865), miscRNA. | -1.24 | 0.007 |
| LOC645693 | XR_017498.2 | PREDICTED: Homo sapiens misc_RNA (LOC645693), miscRNA. | -1.24 | 0.004 |
| TUFT1 | NM_020127.1 | Homo sapiens tuftelin 1 (TUFT1), mRNA. | -1.24 | 0.003 |
| C15orf44 | XM_940546.1 | Homo sapiens chromosome 15 open reading frame 44 (C15orf44), transcript variant 2, mRNA. | -1.24 | 0.001 |
| TXNDC5 | NM_030810.2 | Homo sapiens thioredoxin domain containing 5 (endoplasmic reticulum) (TXNDC5), transcript variant 1, mRNA. | -1.24 | 0.044 |
| RRAS | NM_006270.2 | Homo sapiens related RAS viral (r-ras) oncogene homolog (RRAS), mRNA. | -1.23 | 0.023 |
| AK3L1 | NM_013410.2 | Homo sapiens adenylate kinase 3-like 1 (AK3L1), nuclear gene encoding mitochondrial protein, transcript variant 6, mRNA. | -1.23 | 0.003 |
| ISY1 | NM_020701.1 | Homo sapiens ISY1 splicing factor homolog (S. cerevisiae) (ISY1), mRNA. | -1.23 | 0.044 |
| MFSD3 | NM_138431.1 | Homo sapiens major facilitator superfamily domain containing 3 (MFSD3), mRNA. | -1.23 | 0.008 |
| TUBB2B | NM_178012.3 | Homo sapiens tubulin, beta 2B (TUBB2B), mRNA. | -1.23 | 0.001 |
| YIPF3 | NM_015388.2 | Homo sapiens Yip1 domain family, member 3 (YIPF3), mRNA. | -1.23 | 0.002 |
| LOC642412 | XM_925931.1 | PREDICTED: Homo sapiens similar to matrix-remodelling associated 8 (LOC642412), mRNA. | -1.23 | 0.007 |
| CAPG | NM_001747.2 | Homo sapiens capping protein (actin filament), gelsolin-like (CAPG), mRNA. | -1.23 | 0.012 |
| ATG4B | NM_178326.2 | Homo sapiens ATG4 autophagy related 4 homolog B (S. cerevisiae) (ATG4B), transcript variant 2, mRNA. | -1.23 | 0.022 |
| FTHL11 | NR_002204.1 | Homo sapiens ferritin, heavy polypeptide-like 11 (FTHL11) on chromosome 8. | -1.23 | 0.027 |
| PRMT2 | NM_001535.2 | Homo sapiens protein arginine methyltransferase 2 (PRMT2), transcript variant 2, mRNA. | -1.23 | 0.000 |
| SYTL2 | NM_206930.1 | Homo sapiens synaptotagmin-like 2 (SYTL2), transcript variant d, mRNA. | -1.23 | 0.005 |
| APLP1 | NM_005166.3 | Homo sapiens amyloid beta (A4) precursor-like protein 1 (APLP1), transcript variant 1, mRNA. | -1.23 | 0.016 |
| EDIL3 | NM_005711.3 | Homo sapiens EGF-like repeats and discoidin I-like domains 3 (EDIL3), mRNA. | -1.23 | 0.010 |
| IFITM2 | NM_006435.1 | Homo sapiens interferon induced transmembrane protein 2 (1-8D) (IFITM2), mRNA. | -1.23 | 0.003 |
| LOC647673 | XM_936731.1 | PREDICTED: Homo sapiens similar to Translationally-controlled tumor protein (TCTP) (p23) (Histamine-releasing factor) (HRF) (Fortilin) (LOC647673), mRNA. | -1.23 | 0.008 |
| LOC100131810 | XR_039079.1 | PREDICTED: Homo sapiens misc_RNA (LOC100131810), miscRNA. | -1.23 | 0.005 |
| TCEA2 | NM_003195.4 | Homo sapiens transcription elongation factor A (SII), 2 (TCEA2), transcript variant 1, mRNA. | -1.22 | 0.011 |
| ASS1 | NM_054012.3 | Homo sapiens argininosuccinate synthetase 1 (ASS1), transcript variant 2, mRNA. | -1.22 | 0.003 |
| LOC100130154 | XM_001717333.1 | PREDICTED: Homo sapiens similar to thymosin, beta 10 (LOC100130154), mRNA. | -1.22 | 0.036 |
| LOXL3 | NM_032603.2 | Homo sapiens lysyl oxidase-like 3 (LOXL3), mRNA. | -1.22 | 0.001 |
| ARSA | NM_000487.3 | Homo sapiens arylsulfatase A (ARSA), mRNA. | -1.22 | 0.007 |
| MVP | NM_017458.2 | Homo sapiens major vault protein (MVP), transcript variant 1, mRNA. | -1.22 | 0.007 |
| RPN2 | NM_002951.2 | Homo sapiens ribophorin II (RPN2), mRNA. | -1.22 | 0.004 |
| TRIB3 | NM_021158.3 | Homo sapiens tribbles homolog 3 (Drosophila) (TRIB3), mRNA. | -1.22 | 0.007 |
| LOC651575 | XM_940750.1 | PREDICTED: Homo sapiens similar to DALR anticodon binding domain containing 3 isoform 1 (LOC651575), mRNA. | -1.22 | 0.000 |
| RPL7A | NM_000972.2 | Homo sapiens ribosomal protein L7a (RPL7A), mRNA. | -1.22 | 0.001 |
| LOC652968 | NM_001037666.1 | Homo sapiens hypothetical protein LOC652968 (LOC652968), mRNA. | -1.22 | 0.004 |
| HLA-F | NM_018950.1 | Homo sapiens major histocompatibility complex, class I, F (HLA-F), mRNA. | -1.22 | 0.000 |
| MED16 | NM_005481.2 | Homo sapiens mediator complex subunit 16 (MED16), mRNA. | -1.22 | 0.002 |
| RNH1 | NM_203385.1 | Homo sapiens ribonuclease/angiogenin inhibitor 1 (RNH1), transcript variant 4, mRNA. | -1.22 | 0.002 |
| ITGB4BP | NM_181469.1 | Homo sapiens integrin beta 4 binding protein (ITGB4BP), transcript variant 3, mRNA. | -1.22 | 0.001 |
| RNASEK | NM_001004333.3 | Homo sapiens ribonuclease, RNase K (RNASEK), mRNA. | -1.22 | 0.001 |
| ILK | NM_001014794.1 | Homo sapiens integrin-linked kinase (ILK), transcript variant 2, mRNA. | -1.22 | 0.049 |
| BACE2 | NM_138991.1 | Homo sapiens beta-site APP-cleaving enzyme 2 (BACE2), transcript variant c, mRNA. | -1.22 | 0.001 |
| LOC390354 | XR_000900.1 | PREDICTED: Homo sapiens similar to ribosomal protein L18a; 60S ribosomal protein L18a, transcript variant 36 (LOC390354), misc RNA. | -1.22 | 0.009 |
| MAGED2 | NM_201222.1 | Homo sapiens melanoma antigen family D, 2 (MAGED2), transcript variant 3, mRNA. | -1.22 | 0.005 |
| TSPO | NM_000714.4 | Homo sapiens translocator protein (18kDa) (TSPO), transcript variant PBR, mRNA. | -1.22 | 0.022 |
| HLA-G | NM_002127.3 | Homo sapiens HLA-G histocompatibility antigen, class I, G (HLA-G), mRNA. | -1.22 | 0.002 |
| PPM1M | NM_144641.1 | Homo sapiens protein phosphatase 1M (PP2C domain containing) (PPM1M), mRNA. | -1.21 | 0.001 |
| ASNS | NM_133436.1 | Homo sapiens asparagine synthetase (ASNS), transcript variant 1, mRNA. | -1.21 | 0.028 |
| NNMT | NM_006169.2 | Homo sapiens nicotinamide N-methyltransferase (NNMT), mRNA. | -1.21 | 0.003 |
| FAM162A | NM_014367.3 | Homo sapiens family with sequence similarity 162, member A (FAM162A), mRNA. | -1.21 | 0.047 |
| PCK2 | NM_004563.2 | Homo sapiens phosphoenolpyruvate carboxykinase 2 (mitochondrial) (PCK2), nuclear gene encoding mitochondrial protein, transcript variant 1, mRNA. | -1.21 | 0.001 |
| CAPNS1 | NM_001749.2 | Homo sapiens calpain, small subunit 1 (CAPNS1), transcript variant 1, mRNA. | -1.21 | 0.007 |
| CHCHD10 | NM_213720.1 | Homo sapiens coiled-coil-helix-coiled-coil-helix domain containing 10 (CHCHD10), mRNA. | -1.21 | 0.003 |
| LOC341965 | XR_039164.1 | PREDICTED: Homo sapiens misc_RNA (LOC341965), miscRNA. | -1.21 | 0.049 |
| FYTTD1 | NM_032288.5 | Homo sapiens forty-two-three domain containing 1 (FYTTD1), transcript variant 1, mRNA. | -1.21 | 0.012 |
| DALRD3 | NM_018114.4 | Homo sapiens DALR anticodon binding domain containing 3 (DALRD3), transcript variant 1, mRNA. | -1.21 | 0.001 |
| DACT1 | NM_016651.4 | Homo sapiens dapper, antagonist of beta-catenin, homolog 1 (Xenopus laevis) (DACT1), mRNA. | -1.21 | 0.000 |
| WRNIP1 | NM_020135.2 | Homo sapiens Werner helicase interacting protein 1 (WRNIP1), transcript variant 1, mRNA. | -1.21 | 0.013 |
| VPS28 | NM_016208.2 | Homo sapiens vacuolar protein sorting 28 homolog (S. cerevisiae) (VPS28), transcript variant 1, mRNA. | -1.21 | 0.002 |
| CCS | NM_005125.1 | Homo sapiens copper chaperone for superoxide dismutase (CCS), mRNA. | -1.21 | 0.005 |
| HM13 | NM_030789.2 | Homo sapiens histocompatibility (minor) 13 (HM13), transcript variant 2, mRNA. | -1.21 | 0.032 |
| DDX47 | NM_016355.3 | Homo sapiens DEAD (Asp-Glu-Ala-Asp) box polypeptide 47 (DDX47), transcript variant 1, mRNA. | -1.21 | 0.025 |
| NEDD9 | NM_006403.2 | Homo sapiens neural precursor cell expressed, developmentally down-regulated 9 (NEDD9), transcript variant 1, mRNA. | -1.21 | 0.000 |
| MIB2 | NM_080875.1 | Homo sapiens mindbomb homolog 2 (Drosophila) (MIB2), mRNA. | -1.21 | 0.022 |
| WISP1 | NM_080838.1 | Homo sapiens WNT1 inducible signaling pathway protein 1 (WISP1), transcript variant 2, mRNA. | -1.20 | 0.043 |
| OPLAH | NM_017570.1 | Homo sapiens 5-oxoprolinase (ATP-hydrolysing) (OPLAH), mRNA. | -1.20 | 0.002 |
| CHAC1 | NM_024111.2 | Homo sapiens ChaC, cation transport regulator homolog 1 (E. coli) (CHAC1), mRNA. | -1.20 | 0.002 |
| C10orf61 | NM_001013840.1 | Homo sapiens chromosome 10 open reading frame 61 (C10orf61), transcript variant 1, mRNA. | -1.20 | 0.007 |
| POLD2 | NM_001127218.1 | Homo sapiens polymerase (DNA directed), delta 2, regulatory subunit 50kDa (POLD2), transcript variant 1, mRNA. | -1.20 | 0.006 |
| LMCD1 | NM_014583.2 | Homo sapiens LIM and cysteine-rich domains 1 (LMCD1), mRNA. | -1.20 | 0.013 |
| LOC100130562 | XM_001723702.1 | PREDICTED: Homo sapiens hypothetical protein LOC100130562, transcript variant 1 (LOC100130562), mRNA. | -1.20 | 0.047 |
| CDC42EP5 | NM_145057.2 | Homo sapiens CDC42 effector protein (Rho GTPase binding) 5 (CDC42EP5), mRNA. | -1.20 | 0.007 |
| CEBPD | NM_005195.2 | Homo sapiens CCAAT/enhancer binding protein (C/EBP), delta (CEBPD), mRNA. | -1.20 | 0.005 |
| FASTK | NM_006712.3 | Homo sapiens Fas-activated serine/threonine kinase (FASTK), transcript variant 1, mRNA. | -1.20 | 0.014 |
| LOC100129379 | XR_039592.1 | PREDICTED: Homo sapiens misc_RNA (LOC100129379), miscRNA. | -1.20 | 0.030 |
| PLXNB2 | XM_371474.3 | Homo sapiens plexin B2 (PLXNB2), mRNA. | -1.20 | 0.028 |
| HLA-H | NR_001434.1 | Homo sapiens major histocompatibility complex, class I, H (pseudogene) (HLA-H), non-coding RNA. | -1.20 | 0.010 |
| LOC650132 | XM_939218.1 | PREDICTED: Homo sapiens similar to chromosome 1 open reading frame 80 (LOC650132), mRNA. | -1.20 | 0.017 |
| GPAA1 | NM_003801.2 | Homo sapiens glycosylphosphatidylinositol anchor attachment protein 1 homolog (yeast) (GPAA1), mRNA. | -1.20 | 0.002 |
| MCTS1 | NM_014060.1 | Homo sapiens malignant T cell amplified sequence 1 (MCTS1), mRNA. | -1.20 | 0.043 |
| SLC1A5 | NM_005628.1 | Homo sapiens solute carrier family 1 (neutral amino acid transporter), member 5 (SLC1A5), mRNA. | -1.20 | 0.016 |
| ATF4 | NM_182810.1 | Homo sapiens activating transcription factor 4 (tax-responsive enhancer element B67) (ATF4), transcript variant 2, mRNA. | -1.20 | 0.013 |
| VKORC1 | NM_024006.4 | Homo sapiens vitamin K epoxide reductase complex, subunit 1 (VKORC1), transcript variant 1, mRNA. | -1.20 | 0.015 |
| NOMO3 | NM_001004067.1 | Homo sapiens NODAL modulator 3 (NOMO3), mRNA. | -1.20 | 0.005 |
| MGC87895 | XM_942712.3 | PREDICTED: Homo sapiens similar to ribosomal protein S14 (MGC87895), mRNA. | -1.20 | 0.008 |
| GTF2IP1 | NR_002206.1 | Homo sapiens general transcription factor II, i, pseudogene 1 (GTF2IP1) on chromosome 7. | -1.20 | 0.040 |
| ZWILCH | NR_003105.1 | Homo sapiens Zwilch, kinetochore associated, homolog (Drosophila) (ZWILCH), transcript variant 2, transcribed RNA. | 1.20 | 0.001 |
| EPHA2 | NM_004431.2 | Homo sapiens EPH receptor A2 (EPHA2), mRNA. | 1.20 | 0.001 |
| CKAP2 | NM_018204.2 | Homo sapiens cytoskeleton associated protein 2 (CKAP2), transcript variant 2, mRNA. | 1.20 | 0.012 |
| LOC731049 | NM_014501.1 | PREDICTED: Homo sapiens similar to Ubiquitin-conjugating enzyme E2S (Ubiquitin-conjugating enzyme E2-24 kDa) (Ubiquitin-protein ligase) (Ubiquitin carrier protein) (E2-EPF5) (LOC731049), mRNA. | 1.20 | 0.007 |
| DCBLD2 | NM_080927.3 | Homo sapiens discoidin, CUB and LCCL domain containing 2 (DCBLD2), mRNA. | 1.20 | 0.002 |
| CCDC47 | NM_020198.1 | Homo sapiens coiled-coil domain containing 47 (CCDC47), mRNA. | 1.20 | 0.023 |
| DONSON | NM_145794.1 | Homo sapiens downstream neighbor of SON (DONSON), mRNA. | 1.20 | 0.009 |
| RERE | NM_012102.3 | Homo sapiens arginine-glutamic acid dipeptide (RE) repeats (RERE), transcript variant 1, mRNA. | 1.20 | 0.004 |
| RBBP8 | NM_203291.1 | Homo sapiens retinoblastoma binding protein 8 (RBBP8), transcript variant 2, mRNA. | 1.20 | 0.008 |
| HNRPA1P4 | XM_372050.4 | PREDICTED: Homo sapiens heterogeneous nuclear ribonucleoprotein A1 pseudogene 4 (HNRPA1P4), mRNA. | 1.20 | 0.014 |
| THAP10 | NM_020147.2 | Homo sapiens THAP domain containing 10 (THAP10), mRNA. | 1.20 | 0.001 |
| KIF2C | NM_006845.2 | Homo sapiens kinesin family member 2C (KIF2C), mRNA. | 1.20 | 0.002 |
| HAUS8 | NM_001011699.1 | Homo sapiens HAUS augmin-like complex, subunit 8 (HAUS8), transcript variant 1, mRNA. | 1.20 | 0.001 |
| SMC4 | NM_001002800.1 | Homo sapiens structural maintenance of chromosomes 4 (SMC4), transcript variant 2, mRNA. | 1.20 | 0.002 |
| SLC29A1 | NM_004955.1 | Homo sapiens solute carrier family 29 (nucleoside transporters), member 1 (SLC29A1), nuclear gene encoding mitochondrial protein, transcript variant 4, mRNA. | 1.20 | 0.002 |
| FANCD2 | NM_001018115.1 | Homo sapiens Fanconi anemia, complementation group D2 (FANCD2), transcript variant 2, mRNA. | 1.20 | 0.004 |
| TIMP3 | NM_000362.4 | Homo sapiens TIMP metallopeptidase inhibitor 3 (TIMP3), mRNA. | 1.20 | 0.000 |
| CDC25B | NM_004358.3 | Homo sapiens cell division cycle 25 homolog B (S. pombe) (CDC25B), transcript variant 2, mRNA. | 1.20 | 0.013 |
| STMN1 | NM_005563.3 | Homo sapiens stathmin 1 (STMN1), transcript variant 1, mRNA. | 1.20 | 0.002 |
| VIL2 | NM_003379.3 | Homo sapiens villin 2 (ezrin) (VIL2), mRNA. | 1.20 | 0.010 |
| RAD21 | NM_006265.1 | Homo sapiens RAD21 homolog (S. pombe) (RAD21), mRNA. | 1.20 | 0.001 |
| SGOL1 | NM_001012413.1 | Homo sapiens shugoshin-like 1 (S. pombe) (SGOL1), transcript variant C1, mRNA. | 1.20 | 0.029 |
| TJP2 | NM_201629.1 | Homo sapiens tight junction protein 2 (zona occludens 2) (TJP2), transcript variant 2, mRNA. | 1.20 | 0.000 |
| UBN1 | NM_001079514.1 | Homo sapiens ubinuclein 1 (UBN1), transcript variant 2, mRNA. | 1.20 | 0.003 |
| LMNB2 | NM_032737.2 | Homo sapiens lamin B2 (LMNB2), mRNA. | 1.20 | 0.002 |
| KLF11 | XM_938887.1 | PREDICTED: Homo sapiens Kruppel-like factor 11 (KLF11), mRNA. | 1.20 | 0.002 |
| XPO4 | NM_022459.3 | Homo sapiens exportin 4 (XPO4), mRNA. | 1.20 | 0.014 |
| DDX39 | NM_005804.2 | Homo sapiens DEAD (Asp-Glu-Ala-Asp) box polypeptide 39 (DDX39), mRNA. | 1.20 | 0.001 |
| WDR62 | NM_173636.3 | Homo sapiens WD repeat domain 62 (WDR62), transcript variant 1, mRNA. | 1.21 | 0.021 |
| DIAPH1 | NM_005219.3 | Homo sapiens diaphanous homolog 1 (Drosophila) (DIAPH1), transcript variant 1, mRNA. | 1.21 | 0.008 |
| HMGCR | NM_000859.1 | Homo sapiens 3-hydroxy-3-methylglutaryl-Coenzyme A reductase (HMGCR), mRNA. | 1.21 | 0.001 |
| MNS1 | NM_018365.1 | Homo sapiens meiosis-specific nuclear structural 1 (MNS1), mRNA. | 1.21 | 0.002 |
| BLM | NM_000057.1 | Homo sapiens Bloom syndrome, RecQ helicase-like (BLM), mRNA. | 1.21 | 0.000 |
| DOCK10 | NM_014689.1 | Homo sapiens dedicator of cytokinesis 10 (DOCK10), mRNA. | 1.21 | 0.005 |
| TRIP13 | NM_004237.2 | Homo sapiens thyroid hormone receptor interactor 13 (TRIP13), mRNA. | 1.21 | 0.001 |
| C13orf33 | NM_032849.2 | Homo sapiens chromosome 13 open reading frame 33 (C13orf33), mRNA. | 1.21 | 0.007 |
| C17orf53 | NM_024032.2 | Homo sapiens chromosome 17 open reading frame 53 (C17orf53), mRNA. | 1.21 | 0.001 |
| BUB1 | NM_004336.2 | Homo sapiens BUB1 budding uninhibited by benzimidazoles 1 homolog (yeast) (BUB1), mRNA. | 1.21 | 0.001 |
| MND1 | NM_032117.2 | Homo sapiens meiotic nuclear divisions 1 homolog (S. cerevisiae) (MND1), mRNA. | 1.21 | 0.001 |
| MSN | NM_002444.2 | Homo sapiens moesin (MSN), mRNA. | 1.21 | 0.015 |
| UBE2C | NM_181800.1 | Homo sapiens ubiquitin-conjugating enzyme E2C (UBE2C), transcript variant 3, mRNA. | 1.21 | 0.010 |
| SAFB | NM_002967.2 | Homo sapiens scaffold attachment factor B (SAFB), mRNA. | 1.21 | 0.002 |
| OIP5 | NM_007280.1 | Homo sapiens Opa interacting protein 5 (OIP5), mRNA. | 1.21 | 0.019 |
| C14orf106 | NM_018353.3 | Homo sapiens chromosome 14 open reading frame 106 (C14orf106), mRNA. | 1.21 | 0.002 |
| ERI1 | NM_153332.3 | Homo sapiens exoribonuclease 1 (ERI1), mRNA. | 1.21 | 0.011 |
| SFRP1 | NM_003012.3 | Homo sapiens secreted frizzled-related protein 1 (SFRP1), mRNA. | 1.22 | 0.001 |
| PHF15 | NM_015288.4 | Homo sapiens PHD finger protein 15 (PHF15), mRNA. | 1.22 | 0.002 |
| NCAPG | NM_022346.3 | Homo sapiens non-SMC condensin I complex, subunit G (NCAPG), mRNA. | 1.22 | 0.002 |
| ZW10 | NM_004724.2 | Homo sapiens ZW10, kinetochore associated, homolog (Drosophila) (ZW10), mRNA. | 1.22 | 0.001 |
| MCM8 | NM_032485.4 | Homo sapiens minichromosome maintenance complex component 8 (MCM8), transcript variant 1, mRNA. | 1.22 | 0.000 |
| CDC42EP4 | NM_012121.4 | Homo sapiens CDC42 effector protein (Rho GTPase binding) 4 (CDC42EP4), mRNA. | 1.22 | 0.008 |
| KIF14 | NM_014875.1 | Homo sapiens kinesin family member 14 (KIF14), mRNA. | 1.22 | 0.005 |
| POLQ | NM_199420.2 | Homo sapiens polymerase (DNA directed), theta (POLQ), mRNA. | 1.22 | 0.004 |
| NET1 | NM_005863.2 | Homo sapiens neuroepithelial cell transforming 1 (NET1), transcript variant 1, mRNA. | 1.22 | 0.000 |
| MELK | NM_014791.2 | Homo sapiens maternal embryonic leucine zipper kinase (MELK), mRNA. | 1.22 | 0.005 |
| MXD3 | NM_031300.2 | Homo sapiens MAX dimerization protein 3 (MXD3), mRNA. | 1.22 | 0.034 |
| BIRC5 | NM_001168.2 | Homo sapiens baculoviral IAP repeat-containing 5 (BIRC5), transcript variant 1, mRNA. | 1.22 | 0.002 |
| PCNT | NM_006031.3 | Homo sapiens pericentrin (PCNT), mRNA. | 1.22 | 0.001 |
| FAM168B | NM_001009993.1 | Homo sapiens family with sequence similarity 168, member B (FAM168B), mRNA. | 1.22 | 0.000 |
| CDK2 | NM_001798.2 | Homo sapiens cyclin-dependent kinase 2 (CDK2), transcript variant 1, mRNA. | 1.22 | 0.001 |
| KIF15 | NM_020242.1 | Homo sapiens kinesin family member 15 (KIF15), mRNA. | 1.22 | 0.001 |
| LIG1 | NM_000234.1 | Homo sapiens ligase I, DNA, ATP-dependent (LIG1), mRNA. | 1.22 | 0.000 |
| ROR1 | NM_005012.1 | Homo sapiens receptor tyrosine kinase-like orphan receptor 1 (ROR1), transcript variant 1, mRNA. | 1.22 | 0.018 |
|  | Hs.575583 | 602538216F1 NIH_MGC_59 Homo sapiens cDNA clone IMAGE:4659674 5, mRNA sequence | 1.22 | 0.000 |
| POLA1 | NM_016937.2 | Homo sapiens polymerase (DNA directed), alpha 1, catalytic subunit (POLA1), mRNA. | 1.23 | 0.002 |
| SLC20A1 | NM_005415.3 | Homo sapiens solute carrier family 20 (phosphate transporter), member 1 (SLC20A1), mRNA. | 1.23 | 0.000 |
| IPO11 | NM_016338.3 | Homo sapiens importin 11 (IPO11), mRNA. | 1.23 | 0.003 |
| FOXM1 | NM_021953.2 | Homo sapiens forkhead box M1 (FOXM1), transcript variant 2, mRNA. | 1.23 | 0.009 |
|  | Hs.25318 | Homo sapiens clone 25194 mRNA sequence | 1.23 | 0.001 |
| GOLPH4 | NM_014498.2 | Homo sapiens golgi phosphoprotein 4 (GOLPH4), mRNA. | 1.23 | 0.002 |
| BARD1 | NM_000465.1 | Homo sapiens BRCA1 associated RING domain 1 (BARD1), mRNA. | 1.23 | 0.010 |
| CDC20 | NM_001255.1 | Homo sapiens cell division cycle 20 homolog (S. cerevisiae) (CDC20), mRNA. | 1.23 | 0.001 |
| CDCA8 | NM_018101.2 | Homo sapiens cell division cycle associated 8 (CDCA8), mRNA. | 1.23 | 0.000 |
| PPIL5 | NM_152329.3 | Homo sapiens peptidylprolyl isomerase (cyclophilin)-like 5 (PPIL5), transcript variant 3, mRNA. | 1.23 | 0.024 |
| COBLL1 | NM_014900.3 | Homo sapiens COBL-like 1 (COBLL1), mRNA. | 1.24 | 0.005 |
| HJURP | NM_018410.2 | Homo sapiens Holliday junction recognition protein (HJURP), mRNA. | 1.24 | 0.000 |
| ING3 | NM_019071.2 | Homo sapiens inhibitor of growth family, member 3 (ING3), transcript variant 1, mRNA. | 1.24 | 0.007 |
| E2F7 | NM_203394.1 | Homo sapiens E2F transcription factor 7 (E2F7), mRNA. | 1.24 | 0.001 |
| SPC24 | NM_182513.1 | Homo sapiens SPC24, NDC80 kinetochore complex component, homolog (S. cerevisiae) (SPC24), mRNA. | 1.24 | 0.000 |
| PDGFRA | NM_006206.3 | Homo sapiens platelet-derived growth factor receptor, alpha polypeptide (PDGFRA), mRNA. | 1.24 | 0.000 |
| DEPDC1 | NM_017779.3 | Homo sapiens DEP domain containing 1 (DEPDC1), mRNA. | 1.24 | 0.026 |
|  | Hs.213061 | Homo sapiens cDNA FLJ38536 fis, clone HCHON2001200 | 1.24 | 0.003 |
| TPX2 | NM_012112.4 | Homo sapiens TPX2, microtubule-associated, homolog (Xenopus laevis) (TPX2), mRNA. | 1.24 | 0.000 |
| KNTC1 | NM_014708.3 | Homo sapiens kinetochore associated 1 (KNTC1), mRNA. | 1.24 | 0.001 |
| CCND1 | NM_053056.1 | Homo sapiens cyclin D1 (CCND1), mRNA. | 1.24 | 0.006 |
| ATP2C1 | NM_014382.2 | Homo sapiens ATPase, Ca++ transporting, type 2C, member 1 (ATP2C1), transcript variant 1, mRNA. | 1.24 | 0.000 |
| KIF4A | NM_012310.2 | Homo sapiens kinesin family member 4A (KIF4A), mRNA. | 1.24 | 0.000 |
| KIAA0101 | NM_014736.4 | Homo sapiens KIAA0101 (KIAA0101), transcript variant 1, mRNA. | 1.25 | 0.010 |
| HNRNPC | NM_001077442.1 | Homo sapiens heterogeneous nuclear ribonucleoprotein C (C1/C2) (HNRNPC), transcript variant 3, mRNA. | 1.25 | 0.000 |
| TMEM194A | NM_015257.2 | Homo sapiens transmembrane protein 194A (TMEM194A), transcript variant 2, mRNA. | 1.25 | 0.003 |
| CDCA5 | NM_080668.2 | Homo sapiens cell division cycle associated 5 (CDCA5), mRNA. | 1.25 | 0.003 |
| NUP205 | NM_015135.1 | Homo sapiens nucleoporin 205kDa (NUP205), mRNA. | 1.25 | 0.001 |
| SUPT16H | NM_007192.2 | Homo sapiens suppressor of Ty 16 homolog (S. cerevisiae) (SUPT16H), mRNA. | 1.25 | 0.001 |
| LRP8 | NM_033300.2 | Homo sapiens low density lipoprotein receptor-related protein 8, apolipoprotein e receptor (LRP8), transcript variant 3, mRNA. | 1.25 | 0.001 |
| CEP55 | NM_018131.3 | Homo sapiens centrosomal protein 55kDa (CEP55), mRNA. | 1.25 | 0.002 |
| MLF1IP | NM_024629.2 | Homo sapiens MLF1 interacting protein (MLF1IP), mRNA. | 1.25 | 0.001 |
| NUSAP1 | NM_016359.2 | Homo sapiens nucleolar and spindle associated protein 1 (NUSAP1), transcript variant 2, mRNA. | 1.25 | 0.000 |
| GAS2L3 | NM_174942.1 | Homo sapiens growth arrest-specific 2 like 3 (GAS2L3), mRNA. | 1.25 | 0.004 |
| LMNB1 | NM_005573.2 | Homo sapiens lamin B1 (LMNB1), mRNA. | 1.26 | 0.004 |
| CSE1L | NM_001316.2 | Homo sapiens CSE1 chromosome segregation 1-like (yeast) (CSE1L), mRNA. | 1.26 | 0.001 |
| MCM3 | NM_002388.3 | Homo sapiens minichromosome maintenance complex component 3 (MCM3), mRNA. | 1.26 | 0.003 |
| CCNA2 | NM_001237.2 | Homo sapiens cyclin A2 (CCNA2), mRNA. | 1.26 | 0.001 |
| C11orf82 | NM_145018.2 | Homo sapiens chromosome 11 open reading frame 82 (C11orf82), mRNA. | 1.26 | 0.001 |
| TGFBR3 | NM_003243.2 | Homo sapiens transforming growth factor, beta receptor III (TGFBR3), mRNA. | 1.26 | 0.002 |
| PKMYT1 | NM_182687.1 | Homo sapiens protein kinase, membrane associated tyrosine/threonine 1 (PKMYT1), transcript variant 2, mRNA. | 1.26 | 0.003 |
| EXO1 | NM_003686.3 | Homo sapiens exonuclease 1 (EXO1), transcript variant 1, mRNA. | 1.26 | 0.000 |
| PLK4 | NM_014264.2 | Homo sapiens polo-like kinase 4 (Drosophila) (PLK4), mRNA. | 1.26 | 0.001 |
| LARP4B | NM_015155.1 | Homo sapiens La ribonucleoprotein domain family, member 4B (LARP4B), mRNA. | 1.26 | 0.000 |
| TOP2A | NM_001067.2 | Homo sapiens topoisomerase (DNA) II alpha 170kDa (TOP2A), mRNA. | 1.26 | 0.000 |
| CCNF | NM_001761.1 | Homo sapiens cyclin F (CCNF), mRNA. | 1.26 | 0.000 |
| ANLN | NM_018685.2 | Homo sapiens anillin, actin binding protein (ANLN), mRNA. | 1.26 | 0.003 |
| CCDC15 | NM_025004.1 | Homo sapiens coiled-coil domain containing 15 (CCDC15), mRNA. | 1.27 | 0.001 |
| CDC7 | NM_003503.2 | Homo sapiens cell division cycle 7 homolog (S. cerevisiae) (CDC7), mRNA. | 1.27 | 0.001 |
| RAD51AP1 | NM_006479.2 | Homo sapiens RAD51 associated protein 1 (RAD51AP1), mRNA. | 1.27 | 0.006 |
| UBR7 | NM_018108.2 | Homo sapiens ubiquitin protein ligase E3 component n-recognin 7 (putative) (UBR7), transcript variant 3, mRNA. | 1.27 | 0.001 |
| CKAP2L | NM_152515.2 | Homo sapiens cytoskeleton associated protein 2-like (CKAP2L), mRNA. | 1.27 | 0.000 |
| FEN1 | NM_004111.4 | Homo sapiens flap structure-specific endonuclease 1 (FEN1), mRNA. | 1.27 | 0.002 |
| CENPF | NM_016343.3 | Homo sapiens centromere protein F, 350/400ka (mitosin) (CENPF), mRNA. | 1.28 | 0.003 |
| APP | NM_201414.1 | Homo sapiens amyloid beta (A4) precursor protein (APP), transcript variant 3, mRNA. | 1.28 | 0.004 |
| LOC100132564 | XM_001713808.1 | PREDICTED: Homo sapiens hypothetical protein LOC100132564 (LOC100132564), mRNA. | 1.28 | 0.035 |
| DLGAP5 | NM_014750.3 | Homo sapiens discs, large (Drosophila) homolog-associated protein 5 (DLGAP5), mRNA. | 1.28 | 0.000 |
| CDCA2 | NM_152562.2 | Homo sapiens cell division cycle associated 2 (CDCA2), mRNA. | 1.28 | 0.001 |
| AURKA | NM_198434.1 | Homo sapiens aurora kinase A (AURKA), transcript variant 5, mRNA. | 1.28 | 0.000 |
| DKK1 | NM_012242.2 | Homo sapiens dickkopf homolog 1 (Xenopus laevis) (DKK1), mRNA. | 1.29 | 0.018 |
| PBK | NM_018492.2 | Homo sapiens PDZ binding kinase (PBK), mRNA. | 1.29 | 0.000 |
| STIL | NM_003035.2 | Homo sapiens SCL/TAL1 interrupting locus (STIL), transcript variant 2, mRNA. | 1.29 | 0.001 |
| FBXO5 | NM_012177.2 | Homo sapiens F-box protein 5 (FBXO5), mRNA. | 1.29 | 0.011 |
| POLA2 | NM_002689.2 | Homo sapiens polymerase (DNA directed), alpha 2 (70kD subunit) (POLA2), mRNA. | 1.29 | 0.000 |
| ATAD2 | NM_014109.2 | Homo sapiens ATPase family, AAA domain containing 2 (ATAD2), mRNA. | 1.30 | 0.026 |
| CENPE | NM_001813.2 | Homo sapiens centromere protein E, 312kDa (CENPE), mRNA. | 1.30 | 0.002 |
| KIAA1524 | NM_020890.1 | Homo sapiens KIAA1524 (KIAA1524), mRNA. | 1.30 | 0.002 |
| RRM1 | NM_001033.2 | Homo sapiens ribonucleotide reductase M1 polypeptide (RRM1), mRNA. | 1.30 | 0.000 |
| TAF15 | NM_003487.2 | Homo sapiens TAF15 RNA polymerase II, TATA box binding protein (TBP)-associated factor, 68kDa (TAF15), transcript variant 1, mRNA. | 1.31 | 0.008 |
| CDC45L | NM_003504.3 | Homo sapiens CDC45 cell division cycle 45-like (S. cerevisiae) (CDC45L), mRNA. | 1.31 | 0.000 |
| SMC2 | NM_001042550.1 | Homo sapiens structural maintenance of chromosomes 2 (SMC2), transcript variant 1, mRNA. | 1.31 | 0.000 |
| E2F2 | NM_004091.2 | Homo sapiens E2F transcription factor 2 (E2F2), mRNA. | 1.31 | 0.002 |
| KIF11 | NM_004523.2 | Homo sapiens kinesin family member 11 (KIF11), mRNA. | 1.31 | 0.000 |
| ASPM | NM_018136.2 | Homo sapiens asp (abnormal spindle) homolog, microcephaly associated (Drosophila) (ASPM), mRNA. | 1.32 | 0.001 |
| COPG2 | NM_012133.2 | Homo sapiens coatomer protein complex, subunit gamma 2 (COPG2), mRNA. | 1.33 | 0.005 |
| GINS2 | NM_016095.1 | Homo sapiens GINS complex subunit 2 (Psf2 homolog) (GINS2), mRNA. | 1.33 | 0.001 |
| MCM4 | NM_005914.2 | Homo sapiens minichromosome maintenance complex component 4 (MCM4), transcript variant 1, mRNA. | 1.34 | 0.000 |
| TYMS | NM_001071.1 | Homo sapiens thymidylate synthetase (TYMS), mRNA. | 1.35 | 0.001 |
| NCAPG2 | NM_017760.5 | Homo sapiens non-SMC condensin II complex, subunit G2 (NCAPG2), mRNA. | 1.36 | 0.001 |
| LOC100008588 | NR_003286.1 | Homo sapiens 18S ribosomal RNA (LOC100008588), non-coding RNA. | 1.38 | 0.023 |
| FAM111A | NM_022074.2 | Homo sapiens family with sequence similarity 111, member A (FAM111A), transcript variant 1, mRNA. | 1.38 | 0.000 |
| CCNE2 | NM_057735.1 | Homo sapiens cyclin E2 (CCNE2), transcript variant 2, mRNA. | 1.41 | 0.004 |
| ASF1B | NM_018154.2 | Homo sapiens ASF1 anti-silencing function 1 homolog B (S. cerevisiae) (ASF1B), mRNA. | 1.41 | 0.015 |
| RRM2 | NM_001034.1 | Homo sapiens ribonucleotide reductase M2 polypeptide (RRM2), mRNA. | 1.43 | 0.024 |
| AADACL1 | NM_020792.3 | Homo sapiens arylacetamide deacetylase-like 1 (AADACL1), mRNA. | 1.43 | 0.000 |
| SNHG3-RCC1 | NM_001269.2 | Homo sapiens SNHG3-RCC1 readthrough transcript (SNHG3-RCC1), transcript variant 1, mRNA. | 1.44 | 0.000 |
| KIFC1 | NM_002263.2 | Homo sapiens kinesin family member C1 (KIFC1), mRNA. | 1.45 | 0.001 |
| UHRF1 | NM_013282.2 | Homo sapiens ubiquitin-like with PHD and ring finger domains 1 (UHRF1), transcript variant 1, mRNA. | 1.50 | 0.001 |
| LOC100134364 | XM_001713810.1 | PREDICTED: Homo sapiens hypothetical protein LOC100134364 (LOC100134364), mRNA. | 1.72 | 0.007 |
| LOC100133565 | XM_001724542.1 | PREDICTED: Homo sapiens similar to hCG23738 (LOC100133565), mRNA. | 1.84 | 0.034 |
| LOC100132394 | XM_001713809.1 | PREDICTED: Homo sapiens hypothetical protein LOC100132394 (LOC100132394), mRNA. | 2.00 | 0.009 |
| LOC100008589 | NR_003287.1 | Homo sapiens 28S ribosomal RNA (LOC100008589), non-coding RNA. | 3.20 | 0.003 |
